# Understanding Drivers of Resistance Toward Implementation of Web-Based Self-Management Tools in Routine Cancer Care Among Oncology Nurses: Cross-Sectional Survey Study

**DOI:** 10.2196/14985

**Published:** 2019-12-17

**Authors:** Matthijs de Wit, Mirella Kleijnen, Birgit Lissenberg-Witte, Cornelia van Uden-Kraan, Kobe Millet, Ruud Frambach, Irma Verdonck-de Leeuw

**Affiliations:** 1 Vrije Universiteit Amsterdam Department of Clinical, Neuro- and Developmental Psychology Amsterdam Netherlands; 2 Cancer Center Amsterdam Amsterdam Netherlands; 3 Amsterdam Public Health Research Institute Amsterdam Netherlands; 4 Vrije Universiteit Amsterdam School of Business and Economics Department of Marketing Amsterdam Netherlands; 5 Amsterdam UMC, Vrije Universiteit Amsterdam Department of Epidemiology and Biostatistics Amsterdam Netherlands; 6 Amsterdam UMC, Vrije Universiteit Amsterdam Department of Otolaryngology-Head and Neck Surgery Amsterdam Netherlands

**Keywords:** psycho-oncology, health-related quality of life, self-management, eHealth, implementation science, resistance to innovations

## Abstract

**Background:**

Supporting patients to engage in (Web-based) self-management tools is increasingly gaining importance, but the engagement of health care professionals is lagging behind. This can partly be explained by resistance among health care professionals.

**Objective:**

The aim of this study was to investigate drivers of resistance among oncology nurses toward Web-based self-management tools in cancer care.

**Methods:**

Drawing from previous research, combining clinical and marketing perspectives, and several variables and instruments, we developed the Resistance to Innovation model (RTI-model). The RTI-model distinguishes between passive and active resistance, which can be enhanced or reduced by functional drivers (incompatibility, complexity, lack of value, and risk) and psychological drivers (role ambiguity, social pressure from the institute, peers, and patients). Both types of drivers can be moderated by staff-, organization-, patient-, and environment-related factors. We executed a survey covering all components of the RTI-model on a cross-sectional sample of nurses working in oncology in the Netherlands. Structural equation modeling was used to test the full model, using a hierarchical approach. In total, 2500 nurses were approached, out of which 285 (11.40%) nurses responded.

**Results:**

The goodness of fit statistic of the uncorrected base model of the RTI-model (n=239) was acceptable (χ^2^_1_=9.2; Comparative Fit Index=0.95; Tucker Lewis index=0.21; Root Mean Square Error of Approximation=0.19; Standardized Root Mean Square=0.016). In line with the RTI-model, we found that both passive and active resistance among oncology nurses toward (Web-based) self-management tools were driven by both functional and psychological drivers. Passive resistance toward Web-based self-management tools was enhanced by complexity, lack of value, and role ambiguity, and it was reduced by institutional social pressure. Active resistance was enhanced by complexity, lack of value, and social pressure from peers, and it was reduced by social pressure from the institute and patients. In contrast to what we expected, incompatibility with current routines was not a significant driver of either passive or active resistance. This study further showed that these drivers of resistance were moderated by expertise (*P*=.03), managerial support (*P*=.004), and influence from external stakeholders (government; *P*=.04).

**Conclusions:**

Both passive and active resistance in oncology nurses toward Web-based self-management tools for patients with cancer are driven by functional and psychological drivers, which may be more or less strong, depending on expertise, managerial support, and governmental influence.

## Introduction

In current health care, self-management is increasingly gaining importance. Self-management is defined as “those tasks that individuals undertake to deal with the medical, role, and emotional management of their health condition(s)” [1]. Despite the benefits of self-management [2,3], engagement by patients, as well as health care professionals, is still lagging behind. Self-management support in cancer care is a rather new area and not yet implemented in routine care in many countries and settings. Electronic health (eHealth) might facilitate engagement in self-management. A recent meta-review on eHealth targeting patients with cancer showed positive effects on perceived support, knowledge levels, and information competence [4]. Therefore, moving toward Web-based self-management tools as part of personal health management seems a promising avenue, but to date, the actual use of such tools is also still more an aspiration than a reality. Implementing innovations successfully, such as integrating Web-based self-management tools in clinical processes, requires institutional commitment from the management and buy-in from health care professionals and, ultimately, patients [5]. Resistance toward innovations, such as Web-based self-management, is an important phenomenon that potentially hinders a successful takeoff. In cancer care, there is an important role for oncology nurses in stimulating and supporting self-management behaviors in patients [6-9]. Understanding potential resistance regarding Web-based self-management among oncology nurses is therefore key to successfully integrating the use of Web-based self-management tools in routine cancer care.

Resistance is typically characterized by different levels of intensity. In line with previous research, we differentiate between passive and active resistance. Passive resistance represents a generic, initial attitude to resist an innovation [10], where an individual (ie, the nurse) does not adopt an innovation or postpones the decision until there is a clear reason to change routine care. Active resistance is characterized by a conscious, deliberate decision. This may go beyond the individual’s decision to reject, involving public displays of disapproval and actively encouraging others to resist the innovation as well [11,12]. Nurses may “make or break” a patient’s use of such tools, as patients often regard nurses as authoritative figures; in addition, they are key players in the introduction of Web-based self-management tools [6-9]. Both passive and active resistance may hamper implementation of Web-based self-management tools, but knowledge about drivers of passive and active resistance among oncology nurses is lacking. Therefore, the objective of this study was to investigate the drivers of passive and active resistance among oncology nurses toward Web-based self-management and identify possible factors that may moderate the effect of these drivers. The results of this study are highly relevant in developing future interventions that aim to improve implementation of Web-based self-management tools.

## Methods

### Study Design, Participants, and Recruitment

All participants in this cross-sectional survey study were registered nurses working in oncology care in Dutch health care organizations. On the basis of sample size calculations for structural equation models, the minimum sample size for the base model structure was set at 100 (anticipated effect size: 0.1; desired statistical power level: 0.95; number of latent variables: 2; number of observed variables: 8; probability level: 0.05) [13]. To investigate potential moderating effects, we aimed to include 400 participants. In line with policies regarding studies among health care professionals in the Netherlands, no informed consent, further than regular provision of information about the study, was needed.

Participants were recruited in the second half of 2016 through “V&VN Oncologie,” a professional association for Dutch oncology nurses. All 2500 members were first approached through a call in the monthly email newsletter to fill out an open Web-based version of the survey (data of respondents were protected and only accessible by authorized users: the authors). Respondents filled out the same version of the survey and were presented with a limited number of questions per page. Respondents were asked to answer all questions. If there were blank answers, respondents were prompted to fill out all answers (respondents could still skip and leave an answer blank). Respondents could navigate freely between successive pages before submitting the Web-based survey. To incentivize participation, participants were offered a €10 participation fee and were able to enter a raffle to win a tablet, smartwatch, or gift card. Initial response was lower than anticipated (*n=276,* initial response rate: 11.04%); therefore, a paper version of the survey was subsequently distributed among the attendees of the yearly 2-day conference for nurses working in Dutch oncology, organized by the V&VN Oncology. This conference was attended by V&VN Oncology members who perhaps had already seen the first survey recruitment messages, as well as nonmembers who had not seen the messages yet. Another 400 hard copies of the survey were distributed at the conference, of which 81 were returned (response rate: 20.3%). In total, 357 out of 2500 nurses responded (response rate 14.28%). To prevent multiple entries from the same individual, double entries were removed on the basis of personal identifiable information entered by participants, preserving the first entry. In addition, initially, only respondents who completed the demographics section of the survey were included, leading to a study population of 285 out of 2500 potential participants (response rate: 11.40%).

### Measures

**Figure 1:**
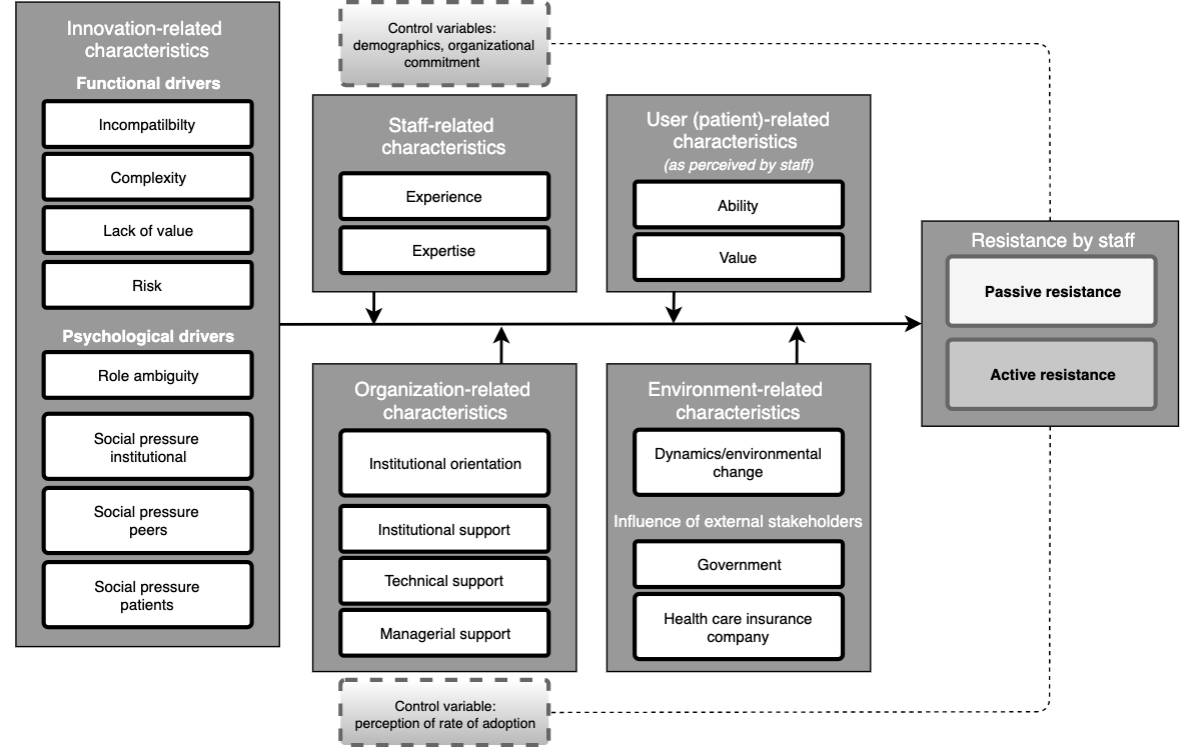
The Resistance to Innovation model.

For this study, the Resistance to Innovation model (RTI-model) was developed and used (Figure 1). The RTI-model draws from theory of innovation resistance, related frameworks in health care, and, especially, the field of implementation science [10,11,14-17]. The outcome to actively or passively resist an innovation can be influenced by functional and psychological drivers. Functional drivers include incompatibility with current routines [18], complexity of the innovation [19], lack of value—especially in comparison with existing alternatives [20,21]—and expected risks about performance and reliability [12,20,22]. Psychological drivers include role ambiguity, which is defined as the lack of clarity on how to incorporate and use an innovation in routine care [23], as well as social pressure [24] from patients, peers, and the organization. The effect of these drivers on resistance may be moderated by staff-, organization-, patient-, and environment-related characteristics. These included experience and expertise in supporting patients in (Web-based) self-management activities (staff related) [23], institutional orientation and support, technical support, managerial support (organization related) [24], ability of patients, as well as value for patients as perceived by nurses (patient related), dynamics of Dutch oncological care [25], and influences of external stakeholders (government and health care insurance companies; environment-related). Several staff-related control variables were included: demographics (age, type of institution, position, counselor position, and years of experience), tendency to comply [26], and organizational commitment [27]. “Perceived rate of adoption” was included as an organization-related control variable (see Figure 1).

To cover all components of the RTI-model (passive and active resistance, functional and psychological drivers of resistance, and moderating and control variables), a 118-item survey was composed, including 34 scales (see Multimedia Appendix 1 for an overview of all items and scales used for each variable. See Multimedia Appendix 2 for the questionnaire). On the basis of psychometric properties, some scales were excluded from further analyses (see Results section and Multimedia Appendix 1 for more information, such as factor loadings). The previously validated multi-item scales were tailored to the context of Web-based self-management and health care institutions in the Netherlands. Most original items were transformed to 5-point Likert scales, ranging from strongly disagree to strongly agree. The draft version of the survey was pretested among 9 nurses using the plus-minus method [28], combined with an interview with debriefing questions. Feedback from this pretest resulted in minor changes, such as reformulation of certain items and answer options, addition of total estimated time needed to fill out the questionnaire, and an example of the concept of self-management for clarification purposes. The electronic survey was thoroughly tested regarding usability and technical functionality by the authors, but this was not part of the pretest. Translation validation was based on consensus among the authors, partially informed by the pretest among 9 nurses.

### Statistical Analyses

A 2-step approach was used to test the RTI-model and assess the fit of the final model [29]. Only complete cases at the base model were included in the analyses of the RTI-model. In the first step, a Confirmatory Factor Analysis (CFA), using Lavaan for R [30], was performed to examine the consistency of all measurement scales for variables with more than 2 items. To purify the measures, factor loadings (factor loading <0.5 and >3.0 compared with the reference factor), fit indices (Comparative Fit Index/Tucker Lewis index [CFI/TLI] ≥0.9; Root Mean Square Error of Approximation [RMSEA] <0.06; Standardized Root Mean Square [SRMR] <0.08), and consensus among the authors were used as decision rules to remove items, to split measurement scales into 2 or more new variables, or to combine separate scales into 1 new variable. These modifications based on the CFA results are reported in the Results section and led to improvements regarding reliability and validity of the set of measures, to perform the next steps in the analysis.

In the second step, structural equation modeling, using Mplus (version 7) [31], was used to test the full model via a hierarchical approach. A base model was estimated first, including all direct effects of functional and psychological drivers on the exogenous constructs’ passive and active resistance. Thereafter, several control variables were added individually to the base model as direct effects on the exogenous constructs. Control variables were modeled uncorrelated with the endogenous constructs, and correlations were added one by one on the basis of modification indices >10. The control variables were “demographics” (modeled as 1 latent variable comprising age, type of institution, position, counselor position [providing psychosocial care], and years of experience), organizational commitment, and perception of rate of adoption. Following this, moderators were investigated in the base model with multi-group comparison, after dividing the moderator in 2 groups by using a median split. Finally, each moderator was added as direct effects to the base model and to each of the models that were corrected for the control variables. The moderators were modeled to be uncorrelated to the endogenous constructs and control variables, and variables were allowed to be correlated on the basis of modification indices >10 to improve the fit of the model.

## Results

### Sample Characteristics

In total, 285 nurses participated, of whom 239 participants filled out all questions. Most participants were female (227/239, 94.4%). Their mean age was 43 years (SD 10.8) and a majority (150/239, 62.8%) were oncology nurses by training. The median years of working experience was 6 years (IQR 2-12 years), and the majority worked on a part-time basis. All sample characteristics are presented in Table 1. Differences between nurses who completed the questionnaire and those who did not are presented in Table 2. Groups differed significantly on training (*P=.*01) and type of hospital (*P=.*02).

**Table 1:** Study sample characteristics.

Variables		Nurses (N=239)
Age (years), mean (SD)		43.3 (10.8)
**Sex, n (%)**		
	Female	227 (94.4)
	Male	12 (5.6)
**Training, n (%)**		
	Nurse	25 (10.5)
	Nurse specialist	64 (26.8)
	Oncology nurse	150 (62.8)
**Counseling, n (%)**		
	Yes	128 (53.6)
	No	111 (46.4)
**Type of hospital, n (%)**		
	University hospital	69 (28.9)
	General teaching hospital	91 (38.1)
	General hospital	54 (22.6)
	Miscellaneous (home care and hospice)	25 (10.5)
**New patients each year, n (%)**		
	0-50	37 (15.5)
	51-100	83 (34.7)
	>100	118 (49.4)
	Missing	1 (0.4)
Years of experience current position, median (IQR)		6 (2-12)
Working hours per week, median (IQR)		32 (26-32)

**Table 2:** Statistics of incomplete cases compared with complete cases.

Variables		Complete cases (N=239)	Incomplete cases (N=46)	*P* value
Age (years), mean (SD)		43.3 (10.8)	44.2 (10.9)	.62
**Sex, n (%)**				.29
	Female	227 (94.4)	41 (89.1)	
	Male	12 (5.6)	4 (8.7)	
	Missing	0 (0.0)	1 (2.2)	
**Training, n (%)**				.01
	Nurse	25 (10.5)	12 (26.1)	
	Nurse specialist	64 (26.8)	8 (17.4)	
	Oncology nurse	150 (62.8)	26 (56.5)	
**Counseling, n (%)**				.26
	Yes	128 (53.6)	20 (43.5)	
	No	111 (46.4)	25 (54.3)	
	Missing	0 (0.0)	1 (2.2)	
**Type of hospital, n (%)**				.02
	University hospital	69 (28.9)	8 (17.4)	
	General teaching hospital	91 (38.1)	15 (32.6)	
	General hospital	54 (22.6)	11 (23.9)	
	Miscellaneous (home care and hospice)	25 (10.5)	12 (26.1)	
**Number of new patients each year, n (%)**				.13
	0-50	37 (15.5)	9 (19.6)	
	51-100	83 (34.7)	9 (19.6)	
	>100	118 (49.4)	28 (60.9)	
	Missing	1 (0.4)	0 (0)	
**Years of experience current position**				.22
	Median	6	8	
	Range	0-36	0-30	
	IQR	2-12	2-14	
**Working hours per week**				.34
	Median	32	30	
	Range	16-40	24-38	
	IQR	26-32	25.5-32	

### Measurement Model Results

#### Confirmatory Factor Analyses

The validity of most scales with more than 2 items was good. For 5 scales, either 1 item was removed or more items were removed: complexity (item 2; functional driver), social pressure from the institution (item 2; psychological driver), experience (item 1 and 3; staff-related moderator), perception of rate of adoption (item 1; control variable of organizational-related moderator), and rejection (item 1; active resistance). Active resistance was initially operationalized on 2 subdimensions—rejection and opposition—but based on the analysis merged into 1 overall variable capturing active resistance. A total of 2 scales were split: dynamics of the environment (item 1 and 3 and item 2 and 4; environment-related moderator) and tendency to comply (item 1 and 2; staff-related control variable). Final factor loadings for each scale are reported in Multimedia Appendix 1, and an overview of all decisions made on the basis of the CFA results.

#### Structural Equation Model

**Figure 2:**
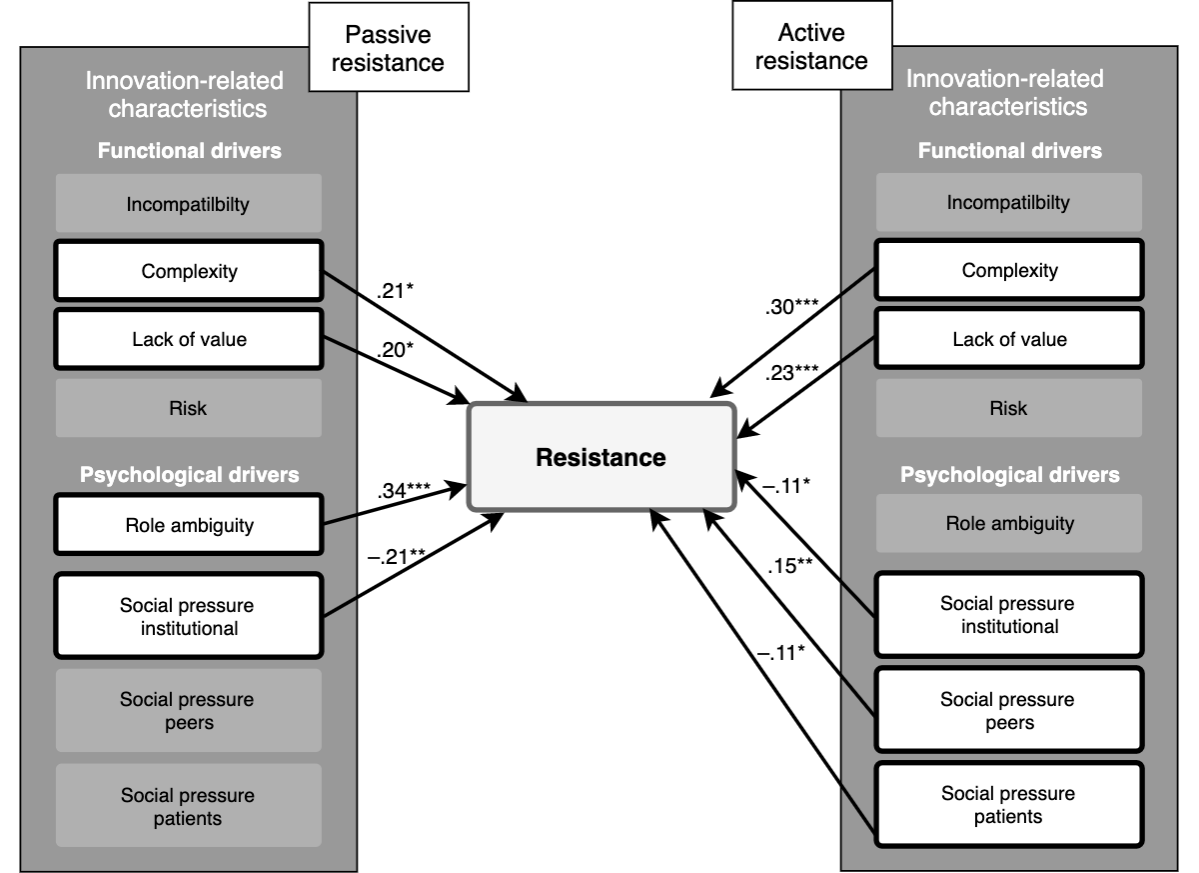
Schematic overview of the effects of functional and psychological drivers on passive and active resistance. *P≤.05, **P≤.01, ***P≤.001.

The goodness of fit statistic of the uncorrected base model (Figure 2) was acceptable [32]—χ^2^_1_=9.2; CFI=0.95; TLI=0.21; RMSEA=0.19; SRMR=0.016; Tables 3 and 4. Passive resistance was enhanced by complexity, lack of value, and role ambiguity, whereas social pressure from the institute significantly reduced passive resistance. Active resistance was enhanced by complexity, lack of value, and social pressure from peers, and this was reduced by social pressure from the institution and from patients. Of the moderators presented in Figure 1, only the significant moderators are discussed, that is, expertise, managerial support, and influence external stakeholders (government; see Tables 3 and 4 and Figure 2). Correcting the models for the control variables produced similar factor loadings and model fits results.

**Table 3:** Standardized factor loadings of functional and psychological drivers on active and passive resistance, including moderator effects: Base model (Model 1), Expertise regarding self-management as moderator (Model 2), Influence from Government as moderator (Model 3), and Managerial support as moderator (Model 4).

Driver		Model 1: Base model (N=239)	Model 2: Expertise		Model 3: Influence from government		Model 4: Managerial support	
			Low (N=110)	High (n=129)	Low (n=104)	High (n=135)	Low (n=126)	High (n=111)
**Passive resistance**								
	Incompatibility	0.035	0.12	−0.035	0.14	−0.053	0.010	0.14
	Complexity	0.21^a^	0.24	0.093	0.048	0.37^b^	0.094	0.39^b^
	Lack of value	0.20^a^	0.44^c^	0.12	0.23	0.13	0.094	0.24^a^
	Risk	0.15	−0.068	0.26^a^	0.064	0.25^a^	0.12	0.19
	Role ambiguity	0.34^c^	0.11	0.39^b^	0.34^b^	0.34^b^	0.31^b^	0.30^b^
	Social pressure: institutional	−0.21^b^	−0.32^c^	−0.12	−0.075	−0.29^b^	−0.073	−0.27^b^
	Social pressure: peers	−0.033	0.006	−0.15	−0.13	0.023	−0.089	0.12
	Social pressure: patients	−0.068	0.076	−0.14	−0.19	0.067	−0.098	−0.020
**Active resistance**								
	Incompatibility	0.031	0.10	−0.001	0.010	0.022	−0.079	0.22
	Complexity	0.30^c^	0.37^c^	0.22^b^	0.18	0.38^c^	0.19^b^	0.44^c^
	Lack of value	0.23^c^	0.41^c^	0.14	0.12	0.26^c^	0.12	0.26^b^
	Risk	0.089	−0.058	0.15^a^	0.17	0.075	0.14^a^	0.088
	Role ambiguity	−0.041	−0.13	−0.022	−0.027	−0.045	0.066	−0.16
	Social pressure: institutional	−0.11^a^	−0.13^a^	−0.11	0.008	−0.15^b^	−0.065	−0.21^b^
	Social pressure: peers	0.15^b^	0.019	0.19^b^	0.012	0.21^c^	0.050	0.30^c^
	Social pressure: patients	−0.11^a^	0.044	−0.21^b^	−0.20^a^	0.010	−0.13^a^	−0.025

^a^*P*≤.05.

^b^*P*≤.01.

^c^*P*≤.001.

**Table 4:** Model information of the different models: Base model (Model 1), Expertise regarding self-management as moderator (Model 2), Influence from Government as moderator (Model 3), and Managerial support as moderator (Model 4).

Model information		Model 1	Model 2	Model 3	Model 4
**Mode fit indices**					
	χ^2^ (*df*)	9.2 (1)	8.4 (2)	6.2 (2)	7.2 (2)
	*P* value	.002^a^	.02^b^	.045^b^	.03^b^
	CFI^c^	0.95	0.95	0.98	0.97
	TLI^d^	0.21	0.22	0.61	0.51
	RMSEA^e^	0.19	0.16	0.13	0.15
	SRMR^f^	0.016	0.016	0.013	0.015
	Multigroup comparison: Wald (*df*=16); *P* value	—^g^	28.21; .03^b^	27.472; .04^b^	34.91; .004^a^
**Residual variances**					
	Passive resistance	0.43	0.32^h^; 0.47^i^	0.43^h^; 0.40^i^	0.46^h^; 0.30^i^
	Active resistance	0.19	0.17^h^; 0.18^i^	0.19^h^; 0.16^i^	0.16^h^; 0.19^i^

^a^*P*≤.01.

^b^*P*≤.05.

^c^CFI: Comparative Fit Index.

^d^TLI: Tucker Lewis index.

^e^RMSEA: Root Mean Square Error of Approximation.

^f^SRMR: Standardized Root Mean Square Residual.

^g^Not applicable.

^h^Low subgroup.

^i^High subgroup.

**Figure 3:**
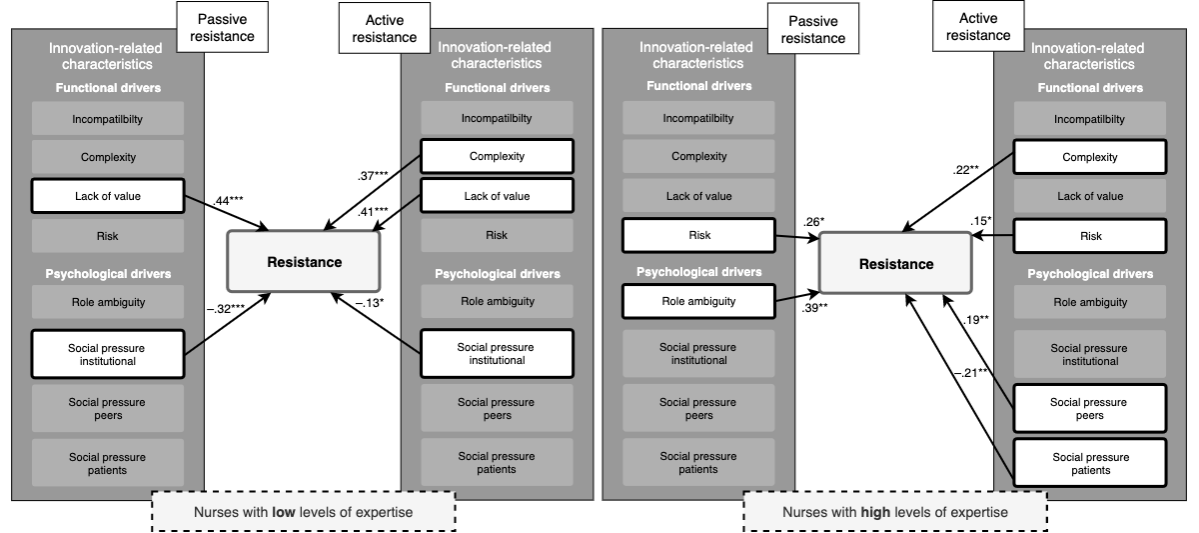
Schematic overview of the moderating effect of expertise among nurses with low (left) and high (right) levels of expertise regarding self-management. *P≤.05, **P≤.01, ***P≤.001.

With regard to possible staff-related moderators, expertise regarding self-management was found to significantly moderate the effect of functional and psychological drivers on resistance (*P=.*03, Table 4), whereas experience was not (*P=.*29). Among nurses with a low level of expertise, lack of value enhanced passive resistance, whereas institutional pressure reduced passive resistance. Complexity and lack of value enhanced active resistance, and institutional pressure reduced active resistance for this group (Figure 3). Among nurses with a high level of expertise (Figure 3), risk and role ambiguity significantly enhanced passive resistance. Complexity, risk, and social pressure from peers significantly enhanced active resistance among nurses with high levels of expertise, whereas pressure from patients reduced active resistance among these nurses.

**Figure 4:**
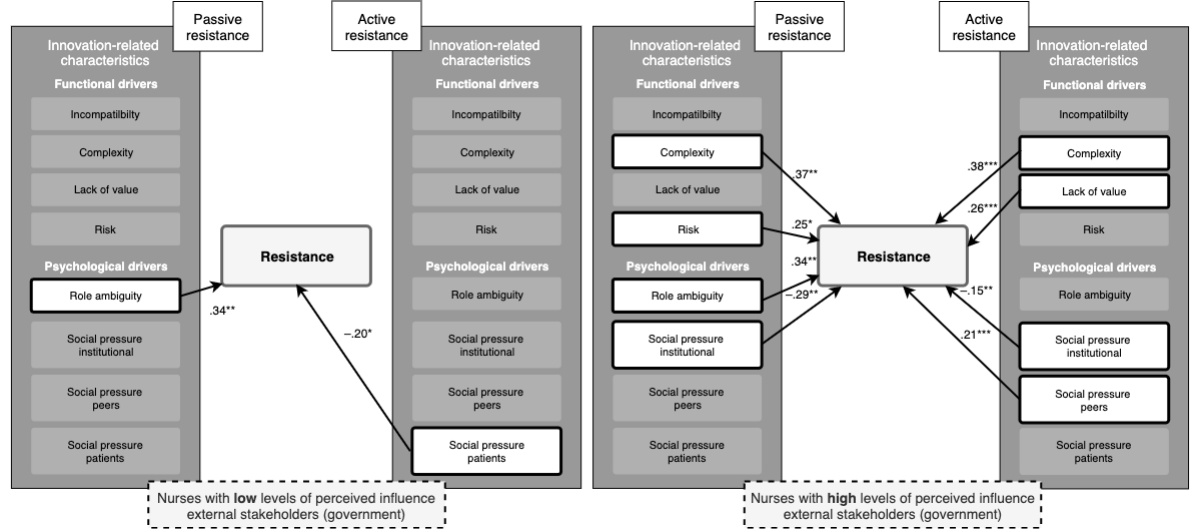
Schematic overview of the moderating effect of perceived influence of external stakeholders (government) among nurses with low (left) and high (right) levels of perceived influence of external stakeholders (government). *P≤.05, **P≤.01, ***P≤.001.

**Figure 5:**
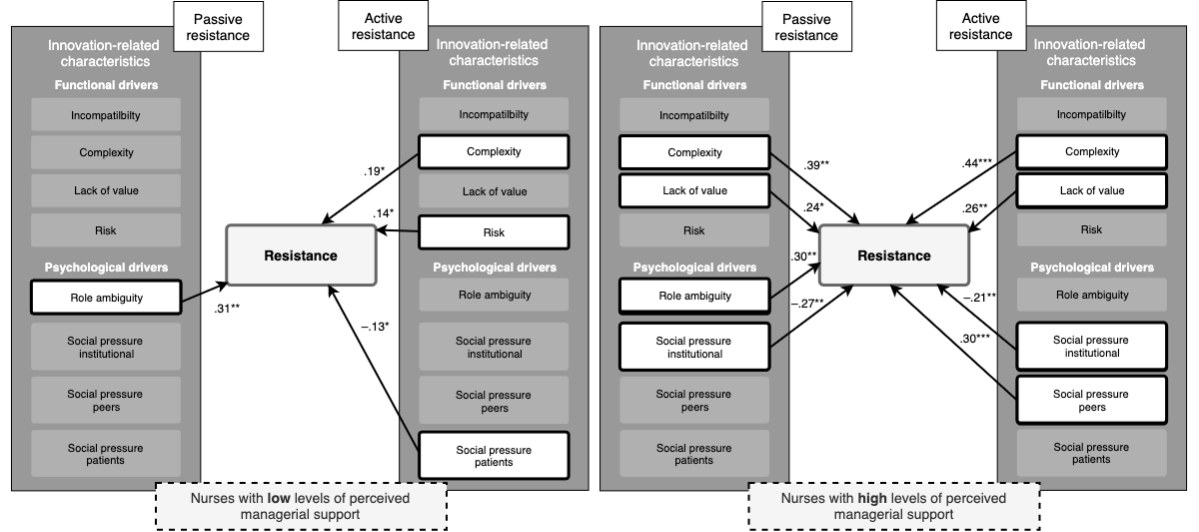
Schematic overview of the moderating effects of managerial support on the effect of functional and psychological drivers on passive and active resistance among nurses with low (left) and high (right) levels of managerial support. *P≤.05, **P≤.01, ***P≤.001.

Perceived influence of external stakeholders (government; environmental moderator) also had a significant moderating effect (*P=.*04). Among nurses who perceived lower levels of influence from the government (Figure 4), role ambiguity significantly enhanced passive resistance, and social pressure from patients significantly reduced active resistance. Among nurses who perceived high levels of influence from the government (Figure 4), complexity, risk, and role ambiguity enhanced passive resistance, whereas social pressure from the institute significantly reduced passive resistance. Among these nurses who perceived high levels of governmental influence, complexity, lack of value, and social pressure from peers significantly enhanced active resistance. Social pressure from the institute significantly reduced active resistance (Figure 4). Another significant moderating effect was found for managerial support (*P=.*004; organizational moderator). Among nurses who perceived lower levels of managerial support (Figure 5), role ambiguity significantly enhanced passive resistance; complexity and risk significantly enhanced active resistance, whereas social pressure from patients significantly reduced active resistance. Among nurses who perceived a high level of managerial support (Figure 5), complexity, lack of value, and role ambiguity were significant drivers of passive resistance, whereas institutional social pressure significantly reduced passive resistance. For this group, complexity, lack of value, and social pressure from peers enhanced active resistance, whereas institutional social pressure significantly reduced active resistance (Figure 5).

## Discussion

### Principal Findings

This study showed that among oncology nurses, passive resistance toward Web-based self-management tools was enhanced by complexity, lack of value, and role ambiguity, and it was reduced by institutional social pressure. Active resistance was enhanced by complexity, lack of value, and social pressure from peers, and it was reduced by social pressure from the institute and patients. In contrast to what we expected, incompatibility with current routines was not a significant driver of either passive or active resistance. This study further showed that these drivers of resistance were moderated by expertise, managerial support, and influence from external stakeholders (government).

Perceived complexity of working with Web-based self-management tools was an important driver of both passive and active resistance among nurses, which is consistent with the view that complexity (related to “ease of use”) is a key factor when evaluating innovations or technology [33,34]. Manufacturers of innovations should therefore pay extra attention to make sure the end user understands the advantage of using the innovation. If the advantages of a service can be clearly conveyed, health care providers are more likely to try it [23] and experience the (lack of) complexity themselves.

Lack of value was a significant driver of both passive and active resistance in this study. When nurses view an innovation to be of little added value to their current way of working or when they already use existing tools, this leads to higher resistance. This effect was also observed in a study among general practitioners (GPs) on referring patients to self-management programs, where a negative evaluation by GPs of advantages offered by such programs created a barrier toward implementation [35]. Another study investigating nurses’ evaluations of new technologies also recognized “advantages offered” versus “the lack thereof” as the most commonly mentioned driver for both enhancing and impeding implementation [36]. However, even when providers recognize the value of an innovation, it can still potentially lead to resistance. A study on the implementation of patient-reported outcome measures (PROMs) reported that providers still voiced concerns, even when they acknowledged the value of those PROMs [37]. In this study, the lack of value was especially important as a driver of passive and active resistance among nurses with a low level of expertise but not among those with a high level of expertise. Nurses with low levels of expertise are likely to have less knowledge about such tools; therefore, they perhaps have difficulty assessing the value of the tool, resulting in a more conservative evaluation that tends to be more toward the negative side. Addressing lack of value can be challenging, but improving the performance of the tool, as well as positioning it differently, might reduce resistance caused by this driver.

Risk was found to enhance passive and active resistance, but it did so only among nurses with a high level of expertise. Uncertainty about the reliability and satisfaction of use with an innovation can lead to postponement of adoption until the adopting user can learn more about it [12]. In addition, when an innovation is perceived as a major change in the way of working, this is associated with higher levels of perceived risk [38,39]. Nurses with a high level of expertise might consider themselves able and qualified and therefore justified to vocalize their opinions and express their resistance when risk reaches a certain threshold. Risk can potentially be addressed through endorsements and testimonials, as well as facilitation of trialing the tool [12].

Role ambiguity was a significant driver of passive (but not active) resistance, especially in nurses with a high level of expertise. This finding may be similar to what we found for the risk driver: because of their abilities, they are more likely to recognize the problematic nature of role ambiguity and consequently express their concerns about the implementation of such an innovation. A study among customers in their evaluation of a new self-service system found that even a positive evaluation could be trumped by role ambiguity, which may lead to the customer not trying the service [23]. This phenomenon was also observed among nurses, where ambiguous role clarity was linked to low research utilization in evidence-based practice [40]. A recent study looking into self-management support among nurses in oncology found low levels of self-reported confidence and actual use regarding “using assistive devices and technology (ie, eHealth) to provide remote guidance” (ie, Web-based self-management tools), perhaps indicating a form of role ambiguity and therefore low levels of use [41]. Greenhalgh et al postulated that new interventions can have a “hard core” (the innovation itself) and a “soft periphery” (the organizational structures and systems required for the full implementation of the innovation) [42]. Role ambiguity may occur, as the innovation lacks a “hard core” [43] or as clear working instructions or role boundaries are lacking. Role ambiguity may therefore also be addressed by providing education and training, as well as contextually relevant education aids (eg, wallet cards with instructions) [23].

Social pressure was a significant driver of both passive and active resistance. Institutional social pressure reduced both types of resistance, especially among nurses with a low level of expertise, which is in line with findings that coercive institutional pressure results in positive intentions in the adoption process [24]. In addition, social pressure from patients was found to reduce active resistance, especially among nurses with a high level of expertise. In contrast, social pressure from peers was found to significantly enhance active resistance, especially among nurses with high levels of expertise. One explanation could perhaps be found in the psychological reactance theory, which suggests that when an individual perceives a message as threatening to his or her ability to enact free behavior (ie, an experienced nurse being told what to do, ie, by peers), the said individual experiences reactance. This could consequently lead to restorative behaviors (ie, resistance), to restore their threatened freedom [44,45].

Incompatibility was not found to be a significant driver of resistance in this study. This is perhaps partially caused by the perception that the usage of self-management tools can be modified to be compatible with current ways of working.

Moderating effects on drivers of resistance were staff related (nurses’ expertise), organization related (managerial support), and environment related (governmental influence). The moderating effect of expertise suggests that social pressure should be used with caution in implementation interventions, as active resistance among nurses with high levels of expertise can only be reduced by social pressure from patients and the institution, but not from peers. This finding sheds light on the commonly used practice of “implementation champions” [46], suggesting that this is not always the straightforward choice; however, more research is needed to understand this process. Managerial support and governmental influence may be viewed as important sources of stimulation. When these are perceived to be high, the effect of social pressure from patients to reduce resistance disappears, whereas the reducing effects of social pressure from patients on resistance remain when this stimulation is perceived as low. Social pressure from the institute only seems effective in reducing passive and active resistance among nurses with low levels of expertise, when managerial support is perceived to be high, or when governmental influence is perceived to be high. This is in line with current views that when implementing an innovation, both executive vision and strategic vision are of key importance (which could be expressed through social pressure from the institute, managerial support, and governmental influence) [47-49].

### Managerial Implications

Implementation interventions that aim to reduce passive resistance would probably be most effective, addressing the complexity, lack of value, risk, and role ambiguity surrounding the innovation. This could involve, for example, facilitating trial, communicating relative advantages in a better manner, offering testimonials, providing clear instructions for working with the innovation, or even by making (small) changes to the innovation itself. The process of reducing passive resistance can be accelerated when the organization as a whole (including management) makes its positive position about the innovation clear. This would mainly be effective for those organizations in which employees have a low level of expertise. In case of high expertise, endorsement from the organization (and management) may not be as effective.

Implementation interventions that aim to reduce active resistance on the contrary (and not primarily passive resistance), should especially address complexity, as well as lack of value and risk. Making (drastic) changes to the innovation itself (besides earlier mentioned activities to better the evaluation of certain drivers) becomes a more viable option to consider with active resistance, considering the potential consequences of active resistance. In the case of active resistance, institutional pressure does not seem to work for people with high expertise, whereas pressure from peers even enhances it. However, pressure from patients does seem to work for the high expertise group; therefore, this can be an effective “pull” toward usage.

### Limitations

Although this study provided valuable first insights into drivers of resistance among nurses toward Web-based self-management tools, there are some limitations that need to be addressed. Initially, role conflict was included as a driver, but the variable was excluded from further analysis because of problems with the measurement properties. Furthermore, those who completed all questions, compared with those who did not, differed significantly on training and type of hospital, which is a possible indication of participation bias. In addition, as there is no information about nonrespondents, potential selection bias cannot be completely ruled out. Moreover, we did not reach the 400 respondents we initially aimed for. Furthermore, in the survey, the participants were given short and concise descriptions of the tools that were subject to potential resistance. This is of course different from real-world experiences in evaluating innovations, where context also plays an important role. In addition, this cross-sectional study put participants in an “evaluation mindset,” with static information for the duration of filling out the survey. Real-world adoption processes are dynamic however, these keep changing over time, when new information becomes available. The cross-sectional design of the study does not allow to make concrete statements on dynamic effects. In addition, general perceptions on innovative concepts, such as the use of Web-based self-management tools in routine care, are also likely to shift over time (ie, to a more accepting mindset). Future studies should therefore aim to use a longitudinal design, operationalizing the examples that are studied closer to what could be encountered in the real world. Furthermore, generalizations about these findings should be made with caution, as processes like these are very context dependent.

This study contributes to better understanding the drivers of passive and active resistance among nurses who are pivotal stakeholders in the implementation of Web-based self-management tools in routine cancer care. The results of this study are highly relevant to health care organizations that aim to implement Web-based self-management tools.

### Conclusions

Passive resistance and active resistance are driven by functional and psychological drivers, and these drivers are moderated by expertise, managerial support, and governmental influence.

## Appendix

Multimedia Appendix 1 Overview of all results and statistics.

Multimedia Appendix 2 Questionnaire used in this study.

## Abbreviations

CFA: Confirmatory Factor Analysis
CFI: Comparative Fit Index
eHealth: electronic health
GP: general practitioner
PROM: patient-reported outcome measure
RMSEA: Root Mean Square Error of Approximation
RTI-model: Resistance to Innovation model
SRMR: Standardized Root Mean Square
TLI: Tucker Lewis index
