# Supplementary material for: Understanding Drivers of Resistance Toward Implementation of Web-Based Self-Management Tools in Routine Cancer Care Among Oncology Nurses: Cross-Sectional Survey Study
Source: J Med Internet Res. 2019 Dec 17;21(12):e14985. doi: 10.2196/14985 (PMC6938592; doi:10.2196/14985)
Supplement: Multimedia Appendix 2 [file jmir_v21i12e14985_app2.pdf]

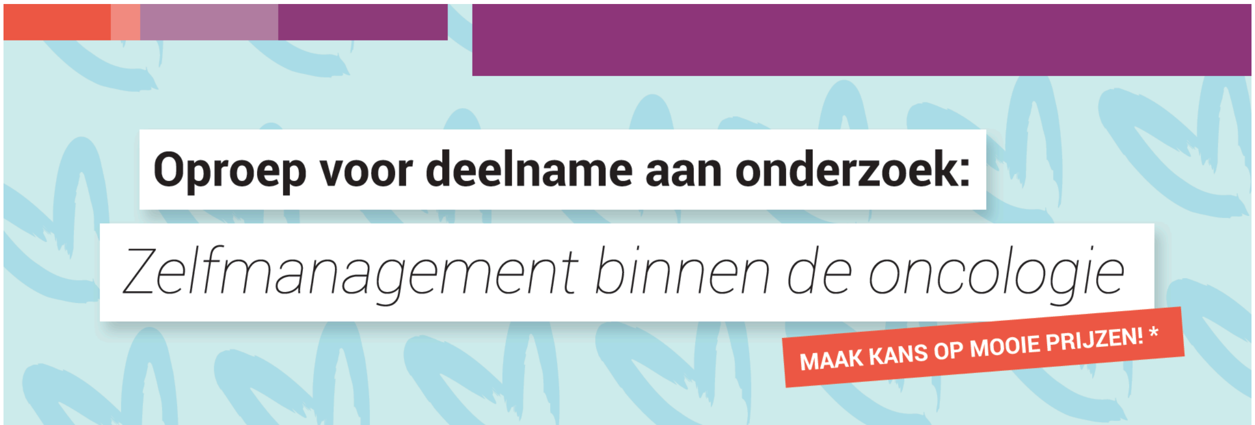

Vragenlijst: Ervaringen en meningen van (oncologisch) verpleegkundigen en verpleegkundig specialisten ten aanzien van online zelfmanagement hulpmiddelen in de zorg

Deze vragenlijst gaat over uw ervaringen met en uw mening over online zelfmanagement hulpmiddelen voor patiënten met kanker. Het onderzoek wordt uitgevoerd in opdracht van de Vrije Universiteit Amsterdam in samenwerking met de V&VN Oncologie. De vragenlijst bestaat uit een algemeen deel (deel A) en een deel over een concreet zelfmanagement hulpmiddel (deel B).

De meerderheid van de mensen doet tussen de 15 en 20 minuten over het invullen van deel A van de vragenlijst. Over deel B doet de meerderheid van de mensen tussen de 5 en 10 minuten.

Uw antwoorden zullen strikt anoniem blijven en worden op geen enkele wijze teruggekoppeld aan de instelling waar u werkzaam bent.

Onder de deelnemers wordt een Apple Watch, een Apple iPad en vier VVV-bonnen t.w.v. 50 euro verloot. Tevens ontvangt u een vergoeding van €10 als dank voor uw medewerking.

Om u te kunnen informeren als u in de prijzen valt, vragen wij om uw e-mailadres aan het einde van de vragenlijst. Als u tevens aanspraak wilt maken op de vergoeding van €10, hebben we naast uw e-mailadres nog aanvullende informatie nodig. Voor het invullen van deze gegevens kunt u het formulier op de laatste pagina's van dit boekje gebruiken.

Uw e-mailadres en eventuele aanvullende informatie worden enkel gebruikt om u te informeren als u in de prijzen bent gevallen en/of de vergoeding van €10 euro aan u uit te keren. Deze gegevens worden op geen enkele wijze gekoppeld aan de door u ingevulde antwoorden.

Bij vragen of problemen kunt u contact opnemen met Matthijs de Wit (promovendus): Matthijs.de.Wit@vu.nl

Deel A1. Uw achtergrond

1. In welk type zorginstelling bent u werkzaam?

- ❍ Academisch ziekenhuis
- ❍ Perifeer opleidingsziekenhuis
- ❍ Perifeer ziekenhuis
- ❍ Thuiszorg
- ❍ Anders, namelijk:

____

2. Wat is uw functie?

- ❍ Verpleegkundige
- ❍ Verpleegkundig specialist
- ❍ Oncologieverpleegkundige
- ❍ Verpleegkundig specialist in opleiding
- ❍ Oncologieverpleegkundige in opleiding
- ❍ Anders, namelijk:

____

3. Sinds welk jaar bent u werkzaam in bovenstaande functie? Sinds:

____

4. Hoeveel uur werkt u per week?

____

5. Heeft u een tijdelijke of een vaste aanstelling?

- ❍ Tijdelijke aanstelling
- ❍ Vaste aanstelling

6. Hoeveel nieuwe patiënten met kanker ziet u per jaar?

- ❍ 1 tot 5 patiënten
- ❍ 6 tot 10 patiënten
- ❍ 11 tot 50 patiënten
- ❍ 51 tot 100 patiënten
- ❍ Meer dan 100 patiënten
- ❍ Geen

7. Heeft u als verpleegkundige (tevens) een consulent functie (spreekuur voor patiënten)?

- ❍ Ja
- ❍ Nee

8. Bij welke organisatie bent u werkzaam? *

____

____

9. Wat is uw leeftijd?

____

10. Wat is uw geslacht?

- ❍ Man
- ❍ Vrouw

*** Uw antwoorden zullen strikt anoniem blijven en worden op geen enkele wijze teruggekoppeld aan de instelling waar u werkzaam bent.**

Deel A2. Uw werk als verpleegkundige

Kunt u voor onderstaande stellingen aangeven in hoeverre u het hiermee eens bent?

1. Wat betreft mijn manier van werken...

	helemaal mee oneens	mee oneens	noch mee oneens, noch mee eens	mee eens	helemaal mee eens
… doe ik wat mijn leidinggevende mij vraagt te doen.	❍	❍	❍	❍	❍
… voer ik de richtlijnen zorgvuldig uit.	❍	❍	❍	❍	❍

2. In de dagelijkse praktijk…

	helemaal mee oneens	mee oneens	noch mee oneens, noch mee eens	mee eens	helemaal mee eens
… maak ik op mijn werk nooit gebruik van het Internet.	❍	❍	❍	❍	❍
… bied ik patiënten vaak online zelfmanagement hulpmiddelen aan.	❍	❍	❍	❍	❍
… maak ik vaak gebruik van de Lastmeter of een vergelijkbaar instrument.	❍	❍	❍	❍	❍

3. In deze vraag staan 5 stellingen. Kunt u voor elke stelling over de uitvoering van uw werk aangeven in hoeverre u het hiermee eens bent?

	helemaal mee oneens	mee oneens	noch mee eens, noch mee oneens	mee eens	helemaal mee eens
Ik geef mijn patiënten meer inzicht in kwaliteit van leven en het verloop hiervan over de tijd.	❍	❍	❍	❍	❍
Ik informeer mijn patiënten over klachten en de samenhang hiertussen.	❍	❍	❍	❍	❍
Ik voorzie mijn patiënten van advies op probleemgebieden.	❍	❍	❍	❍	❍
Ik raad mijn patiënten passende hulpbronnen aan.	❍	❍	❍	❍	❍
Ik stel mijn patiënten in staat om zelfstandig aan het herstel te werken.	❍	❍	❍	❍	❍

4. In deze vraag staan 5 stellingen. Kunt u voor elke stelling aangeven hoe belangrijk u dit vindt om uw werk als zorgprofessional goed uit te voeren?

	helemaal niet belangrijk	niet belangrijk	noch belangrijk, noch onbelangrijk	belangrijk	heel belangrijk
Ik geef mijn patiënten meer inzicht in kwaliteit van leven en het verloop hiervan over de tijd.	❍	❍	❍	❍	❍
Ik informeer mijn patiënten over klachten en de samenhang hiertussen.	❍	❍	❍	❍	❍
Ik voorzie mijn patiënten van advies op probleemgebieden.	❍	❍	❍	❍	❍
Ik raad mijn patiënten passende hulpbronnen aan.	❍	❍	❍	❍	❍
Ik stel mijn patiënten in staat om zelfstandig aan het herstel te werken.	❍	❍	❍	❍	❍

5. In hoeverre bent u het eens met de volgende uitspraken die betrekking hebben op uw houding ten aanzien van technologie en online toepassingen?

	helemaal mee oneens	mee oneens	noch mee oneens, noch mee eens	mee eens	helemaal mee eens
Als ik iets zou horen over technologie en online toepassingen, zou ik zoeken naar manieren om deze uit te proberen.	❍	❍	❍	❍	❍
Ik ben normaal gesproken één van de eersten die nieuwe technologie en online toepassingen gebruikt.	❍	❍	❍	❍	❍
Ik sta open voor nieuwe technologie en online toepassingen.	❍	❍	❍	❍	❍

6. Zijn er, momenteel of binnen afzienbare tijd, veranderingen waar u mee te maken heeft (zoals reorganisatie, fusie, bezuinigingen, personeelsverloop, innovaties)?

❍ Ja
❍ Nee

Zo ja, welke veranderingen?

7. Geef aan hoe vaak de volgende aspecten in uw beleving veranderen binnen de oncologische zorg (in het algemeen in Nederland):

	vrijwel nooit				zeer vaak
Producten en diensten van andere ziekenhuizen, organisaties en bedrijven	❍	❍	❍	❍	❍
Behoeftes van patiënten	❍	❍	❍	❍	❍
Technologische innovaties	❍	❍	❍	❍	❍
Richtlijnen	❍	❍	❍	❍	❍

8. De volgende stellingen verwijzen naar de zorginstelling waar u werkt. Kunt u aangeven in hoeverre u het hiermee eens bent?

	helemaal mee oneens	mee oneens	noch mee oneens, noch mee eens	mee eens	helemaal mee eens
Ik ervaar de problemen van deze zorginstelling als mijn eigen problemen.	❍	❍	❍	❍	❍
Ik heb het gevoel dat ik echt bij deze zorginstelling hoor.	❍	❍	❍	❍	❍
Ik voel me emotioneel gehecht aan deze zorginstelling.	❍	❍	❍	❍	❍
Ik voel me als 'een deel van de familie' in deze organisatie.	❍	❍	❍	❍	❍
Deze zorginstelling betekent veel voor mij.	❍	❍	❍	❍	❍
Binnen mijn zorginstelling worden innovaties alleen binnen pilottrajecten ingezet en volgt geen structurele implementatie.	❍	❍	❍	❍	❍
Binnen mijn zorginstelling worden over het algemeen innovaties snel als onderdeel van reguliere processen geïmplementeerd.	❍	❍	❍	❍	❍
Mijn zorginstelling is zeer dynamisch en biedt ruimte voor een ondernemersgeest. Mensen zijn bereid hun nek uit te steken en risico's te nemen.	❍	❍	❍	❍	❍
Toewijding aan innovatie en ontwikkeling zijn kernelementen van mijn zorginstelling. Er wordt benadrukt voorloper te zijn met producten en diensten.	❍	❍	❍	❍	❍
Binnen mijn zorginstelling is er een nadruk op groei via de ontwikkeling van nieuwe ideeën. Innovaties zijn belangrijk.	❍	❍	❍	❍	❍

9. In hoeverre bent u het eens met de volgende stellingen die gaan over uw werk als verpleegkundige?

	helemaal mee oneens	mee oneens	noch mee oneens, noch mee eens	mee eens	helemaal mee eens
Ik kan kalm blijven wanneer ik geconfronteerd word met moeilijkheden in mijn werk, omdat ik kan terugvallen op mijn vaardigheden.	❍	❍	❍	❍	❍
Wanneer ik geconfronteerd word met een probleem in mijn werk, dan vind ik meestal verschillende oplossingen.	❍	❍	❍	❍	❍
Wat er ook gebeurt in mijn werk, ik kan het gewoonlijk wel aan.	❍	❍	❍	❍	❍
De ervaringen die ik in het verleden in mijn werk heb opgedaan, hebben me goed voorbereid op mijn werk in de toekomst.	❍	❍	❍	❍	❍
Ik haal de doelstellingen die ik aan mezelf stel in mijn werk.	❍	❍	❍	❍	❍
Ik heb voldoende kennis en ervaring om de eisen van mijn werk het hoofd te bieden.	❍	❍	❍	❍	❍

Deel A3. Online zelfmanagement hulpmiddelen

De gezondheidszorg is in ontwikkeling en er is steeds meer aandacht voor online zelfmanagement. Zelfmanagement wordt gedefinieerd als "*those tasks that individuals undertake to deal with the medical, role, and emotional management of their health condition(s)*". Online zelfmanagement richt zich op het verhogen van eigen effectiviteit en empowerment van de patiënt met behulp van een online omgeving. Met andere woorden, het nemen van de eigen regie gedurende en na de behandeling van kanker. Er zijn al veel online hulpmiddelen ter ondersteuning van zelfmanagement beschikbaar.

Een voorbeeld hiervan is kanker.nl, een digitaal platform met betrouwbare informatie over kanker, behandelingen en gevolgen. Deelnemers kunnen blogs van andere deelnemers lezen en deze ook zelf schrijven. Daarnaast zijn er verschillende online discussiegroepen waar patiënten aan kunnen deelnemen. Een ander voorbeeld is het Oncokompas. Het Oncokompas brengt middels vragenlijsten in kaart hoe het gaat met de patiënt (meten), geeft een persoonlijk advies op maat (weten) en biedt een overzicht met passende opties voor ondersteunende zorg bij kanker (doen).

Ook keuzehulpen behoren tot online zelfmanagement hulpmiddelen. Keuzehulpen zijn bedoeld om patiënten te helpen bij het maken van de keuze welke behandeling het beste bij hen past (bijvoorbeeld bij de keuze voor een behandeling voor prostaatkanker of bij de keuze tussen een borstbesparende operatie of een borstamputatie bij borstkanker).

Dit onderdeel van de vragenlijst gaat over uw visie op online zelfmanagement hulpmiddelen, de ondersteuning binnen uw zorginstelling, en het gebruik ervan.

1. Kunt u aangeven in hoeverre u het eens bent met de volgende stellingen die gaan over de zorginstelling waar u werkt?

	helemaal mee oneens	mee oneens	noch mee oneens, noch mee eens	mee eens	helemaal mee eens
Ik word door mijn leidinggevende aangemoedigd om online zelfmanagement hulpmiddelen voor patiënten te gebruiken bij mijn werk.	❍	❍	❍	❍	❍
Mijn leidinggevende ondersteunt uitdrukkelijk het gebruik van online zelfmanagement hulpmiddelen voor patiënten.	❍	❍	❍	❍	❍
Mijn leidinggevende gelooft oprecht in de voordelen van online zelfmanagement hulpmiddelen voor patiënten.	❍	❍	❍	❍	❍
Ik weet waar ik terecht kan als ik ondersteuning nodig heb bij online zelfmanagement hulpmiddelen voor patiënten.	❍	❍	❍	❍	❍
In mijn zorginstelling hebben we goede technische ondersteuning bij online zelfmanagement hulpmiddelen voor patiënten.	❍	❍	❍	❍	❍
We hebben uitgebreide ondersteuning om problemen op te lossen met online zelfmanagement hulpmiddelen voor patiënten.	❍	❍	❍	❍	❍
Binnen mijn zorginstelling wordt de invoering van op de patiënt gerichte innovaties centraal gecoördineerd.	❍	❍	❍	❍	❍
Binnen mijn zorginstelling krijg ik als zorgverlener de ruimte om op de patiënt gerichte innovaties te introduceren.	❍	❍	❍	❍	❍

2. Kunt u aangeven in hoeverre u het eens bent met de volgende stellingen over online zelfmanagement hulpmiddelen?

	helemaal mee oneens	mee oneens	noch mee oneens, noch mee eens	mee eens	helemaal mee eens
Ik verwacht goed in staat te zijn / ik ben goed in staat om patiënten te ondersteunen als het gaat om online zelfmanagement hulpmiddelen bij kanker.	❍	❍	❍	❍	❍
Ik heb vertrouwen in mijn vaardigheden om het gebruik van online zelfmanagement hulpmiddelen bij patiënten te ondersteunen.	❍	❍	❍	❍	❍
Het ondersteunen van patiënten ten aanzien van online zelfmanagement hulpmiddelen past goed binnen mijn mogelijkheden.	❍	❍	❍	❍	❍
Ik heb <u>niet</u> het gevoel dat ik gekwalificeerd ben om patiënten bij te staan in het gebruik van online zelfmanagement hulpmiddelen.	❍	❍	❍	❍	❍

3. Er zijn diverse online zelfmanagement hulpmiddelen in ontwikkeling die in de komende jaren zullen worden geïntroduceerd. Kunt u aangeven in hoeverre u het eens bent met de volgende stellingen over de introductie van <u>nieuwe of net geïntroduceerde</u> online zelfmanagement hulpmiddelen in uw organisatie?

	helemaal mee oneens	mee oneens	noch mee oneens, noch mee eens	mee eens	helemaal mee eens
Ik denk dat het moeilijk zal zijn om het aanbieden van dergelijke online zelfmanagement hulpmiddelen in mijn bestaande werkwijze in te passen.	❍	❍	❍	❍	❍
Het aanbieden van dergelijke online zelfmanagement hulpmiddelen is in strijd met mijn huidige manier van werken.	❍	❍	❍	❍	❍
Ik denk dat het aanbieden van dergelijke online zelfmanagement hulpmiddelen goed past in mijn dagelijkse routine.	❍	❍	❍	❍	❍
Dergelijke online zelfmanagement hulpmiddelen lijken mij moeilijk om te gebruiken.	❍	❍	❍	❍	❍
Het lijkt mij makkelijk om dergelijke online zelfmanagement hulpmiddelen te laten doen wat ik wil.	❍	❍	❍	❍	❍
Dergelijke online zelfmanagement hulpmiddelen lijken mij lastig te begrijpen.	❍	❍	❍	❍	❍
Ik moet erg nadenken bij het gebruik van dergelijke online zelfmanagement hulpmiddelen.	❍	❍	❍	❍	❍
Ik heb er vertrouwen in dat dergelijke online zelfmanagement hulpmiddelen hetgeen zullen kunnen doen zoals beschreven.	❍	❍	❍	❍	❍
Ik ben er niet zeker van dat dergelijke online zelfmanagement hulpmiddelen naar tevredenheid zullen werken.	❍	❍	❍	❍	❍
Ik betwijfel of dergelijke online zelfmanagement hulpmiddelen betrouwbaar zijn in gebruik.	❍	❍	❍	❍	❍

4. Vervolgvragen over <u>nieuwe</u> online zelfmanagement hulpmiddelen

	helemaal mee oneens	mee oneens	noch mee oneens, noch mee eens	mee eens	helemaal mee eens
Ik voel me onzeker over hoe ik nieuwe online zelfmanagement hulpmiddelen effectief kan inzetten in mijn werk.	❍	❍	❍	❍	❍
Ik twijfel over hoe ik nieuwe online zelfmanagement hulpmiddelen op de juiste wijze moet gebruiken.	❍	❍	❍	❍	❍
Ik weet niet zeker wat van mij zal worden verwacht als ik nieuwe online zelfmanagement hulpmiddelen in zal zetten in mijn werk.	❍	❍	❍	❍	❍
Ik denk dat de stappen in het proces om nieuwe online zelfmanagement hulpmiddelen te gebruiken mij niet duidelijk zullen zijn.	❍	❍	❍	❍	❍
Ik verwacht dat er alleen vage aanwijzingen zullen zijn over het gebruik van nieuwe online zelfmanagement hulpmiddelen.	❍	❍	❍	❍	❍
Nieuwe online zelfmanagement hulpmiddelen bieden geen voordelen die al niet door andere, soortgelijke oplossingen geboden worden.	❍	❍	❍	❍	❍
Nieuwe online zelfmanagement hulpmiddelen lossen problemen op die ook door bestaande tools kunnen worden opgelost.	❍	❍	❍	❍	❍

5. Vervolgvragen over nieuwe of net geïntroduceerde online zelfmanagement hulpmiddelen

	helemaal mee oneens	mee oneens	noch mee oneens, noch mee eens	mee eens	helemaal mee eens
Ik verwacht dat mijn patiënten vinden dat we dergelijke online zelfmanagement hulpmiddelen zouden moeten aanbieden.	❍	❍	❍	❍	❍
Ik denk dat er patiënten zijn die er van overtuigd zijn dat we dergelijke online zelfmanagement hulpmiddelen moeten aanbieden.	❍	❍	❍	❍	❍
Ik denk dat mijn collega's vinden dat we dergelijke online zelfmanagement hulpmiddelen zouden moeten aanbieden.	❍	❍	❍	❍	❍
Ik denk dat de meeste verpleegkundigen vinden dat we dergelijke online zelfmanagement hulpmiddelen moeten aanbieden.	❍	❍	❍	❍	❍
Ik verwacht dat verpleegkundigen druk zullen uitoefenen om dergelijke online zelfmanagement hulpmiddelen aan te bieden.	❍	❍	❍	❍	❍

6. Vervolgvragen over nieuwe of net geïntroduceerde online zelfmanagement hulpmiddelen

	helemaal mee oneens	mee oneens	noch mee oneens, noch mee eens	mee eens	helemaal mee eens
Op dit moment overweeg ik nog niet om dergelijke online zelfmanagement hulpmiddelen in te zetten.	❍	❍	❍	❍	❍
Ik stel mijn beslissing over het gebruik van dergelijke online zelfmanagement hulpmiddelen op dit moment nog even uit.	❍	❍	❍	❍	❍
Ik ben geïnteresseerd in het gebruik van dergelijke online zelfmanagement hulpmiddelen, maar ik ga het op dit moment nog niet inzetten.	❍	❍	❍	❍	❍
Het is zeer onwaarschijnlijk dat ik dergelijke online zelfmanagement hulpmiddelen in de nabije toekomst ga inzetten.	❍	❍	❍	❍	❍
Dergelijke online zelfmanagement hulpmiddelen zijn niets voor mij.	❍	❍	❍	❍	❍
Ik ga dergelijke online zelfmanagement hulpmiddelen niet inzetten.	❍	❍	❍	❍	❍

U heeft nu 75% van de vragenlijst ingevuld.

7. Vervolgvragen over <u>nieuwe of net geïntroduceerde</u> online zelfmanagement hulpmiddelen

	helemaal mee oneens	mee oneens	noch mee oneens, noch mee eens	mee eens	helemaal mee eens
Ik zal negatief zijn tegen andere mensen over dergelijke online zelfmanagement hulpmiddelen.	❍	❍	❍	❍	❍
Ik zou andere mensen aanraden dergelijke online zelfmanagement hulpmiddelen niet in te zetten als ze het mij zouden vragen.	❍	❍	❍	❍	❍
Ik zou collega's en bekenden beïnvloeden om dergelijke online zelfmanagement hulpmiddelen niet in te zetten.	❍	❍	❍	❍	❍

8. Vervolgvragen over <u>nieuwe of net geïntroduceerde</u> online zelfmanagement hulpmiddelen

	helemaal mee oneens	mee oneens	noch mee oneens, noch mee eens	mee eens	helemaal mee eens
Ik denk dat mijn zorginstelling vindt dat we dergelijke online zelfmanagement hulpmiddelen zouden moeten aanbieden.	❍	❍	❍	❍	❍
Wellicht verliest mijn zorginstelling een deel van de markt als ze dergelijke online zelfmanagement hulpmiddelen niet aanbieden.	❍	❍	❍	❍	❍
Ik denk dat het ziekenhuismanagement er van overtuigd is dat we dergelijke online zelfmanagement hulpmiddelen moeten aanbieden.	❍	❍	❍	❍	❍
Ik verwacht dat het ziekenhuismanagement druk gaat uitoefenen om dergelijke online zelfmanagement hulpmiddelen aan te bieden.	❍	❍	❍	❍	❍

9. In hoeverre bent u het eens met de volgende uitspraken die betrekking hebben op verzekeraars, de overheid en online zelfmanagement hulpmiddelen?

	helemaal mee oneens	mee oneens	noch mee oneens, noch mee eens	mee eens	helemaal mee eens
Ik denk dat de ziektekostenverzekeraars ervan overtuigd zijn dat we online zelfmanagement hulpmiddelen moeten aanbieden.	❍	❍	❍	❍	❍
Ik verwacht dat ziektekostenverzekeraars druk zullen uitoefenen om online zelfmanagement hulpmiddelen aan te bieden.	❍	❍	❍	❍	❍
Ik denk dat de overheid er van overtuigd is dat we online zelfmanagement hulpmiddelen moeten aanbieden.	❍	❍	❍	❍	❍
Ik verwacht dat de overheid druk zal uitoefenen om online zelfmanagement hulpmiddelen aan te bieden.	❍	❍	❍	❍	❍

10. In hoeverre bent u het eens met de volgende uitspraken die betrekking hebben op patiënten en online zelfmanagement hulpmiddelen?

Ik verwacht dat het gebruik van online zelfmanagement hulpmiddelen door de patiënt ...

	helemaal mee oneens	mee oneens	noch mee oneens, noch mee eens	mee eens	helemaal mee eens
… ertoe zal leiden dat mijn zorginstelling meer kosten moet maken voor de inzet van paramedische of psychosociale zorg.	❍	❍	❍	❍	❍
… ertoe zal leiden dat ik meer tijd kwijt ben per patiënt.	❍	❍	❍	❍	❍
... ten koste gaat van mijn aandacht voor de patiënt.	❍	❍	❍	❍	❍

11. Ik verwacht dat het gebruik van online zelfmanagement hulpmiddelen ertoe zal leiden dat patiënten…

	helemaal mee oneens	mee oneens	noch mee oneens, noch mee eens	mee eens	helemaal mee eens
... te veel informatie ontvangen.	❍	❍	❍	❍	❍
... verward raken.	❍	❍	❍	❍	❍
... angstig worden.	❍	❍	❍	❍	❍
... te veel bezig zullen zijn met hun ziekte.	❍	❍	❍	❍	❍
... onbedoeld bewust worden van problematiek die niet bij hun speelt.	❍	❍	❍	❍	❍

12. Vervolgvragen patiënten en online zelfmanagement hulpmiddelen

	helemaal mee oneens	mee oneens	noch mee oneens, noch mee eens	mee eens	helemaal mee eens
Ik verwacht dat mijn patiënten geen behoefte zullen hebben aan online zelfmanagement hulpmiddelen.	❍	❍	❍	❍	❍
Ik denk dat er al genoeg van dit soort dergelijke toepassingen beschikbaar zijn.	❍	❍	❍	❍	❍
Ik verwacht dat het merendeel van de patiënten geen gebruik zal maken van online zelfmanagement hulpmiddelen als ik deze aan zou bieden.	❍	❍	❍	❍	❍

13. Ik verwacht dat online zelfmanagement hulpmiddelen...

	helemaal mee oneens	mee oneens	noch mee oneens, noch mee eens	mee eens	helemaal mee eens
... niet geschikt zijn voor mijn patiëntenpopulatie.	❍	❍	❍	❍	❍
... te belastend zijn voor mijn patiënten vanwege de vragenlijsten die al vanuit mijn zorginstelling worden afgenomen.	❍	❍	❍	❍	❍
... te belastend zijn voor mijn patiënten vanwege hun ziekte en de bijbehorende behandeling.	❍	❍	❍	❍	❍

14. Stel dat nieuwe online zelfmanagement hulpmiddelen vandaag zouden worden geïntroduceerd in uw zorginstelling:

	zeer onwaarschijnlijk	onwaarschijnlijk	noch onwaarschijnlijk, noch waarschijnlijk	waarschijnlijk	zeer waarschijnlijk
Hoe waarschijnlijk is het dat u dergelijke online zelfmanagement hulpmiddelen zou aanbieden aan patiënten?	❍	❍	❍	❍	❍
Hoe waarschijnlijk is het dat u dit gaat doen binnen drie maanden?	❍	❍	❍	❍	❍
En binnen een jaar?	❍	❍	❍	❍	❍

Deel B. Het Oncokompas$^{2.0}$

Het Oncokompas wordt momenteel landelijk geïmplementeerd in 59 Nederlandse ziekenhuizen en zal worden vergoed uit de basisverzekering. Het Oncokompas bestaat uit de componenten Meten, Weten en Doen. Dit is op de volgende manier uitgewerkt:

De zorgverlener nodigt de patiënt uit. De patiënt ontvangt vervolgens automatisch een e-mail met unieke link om zelfstandig een account aan te maken.

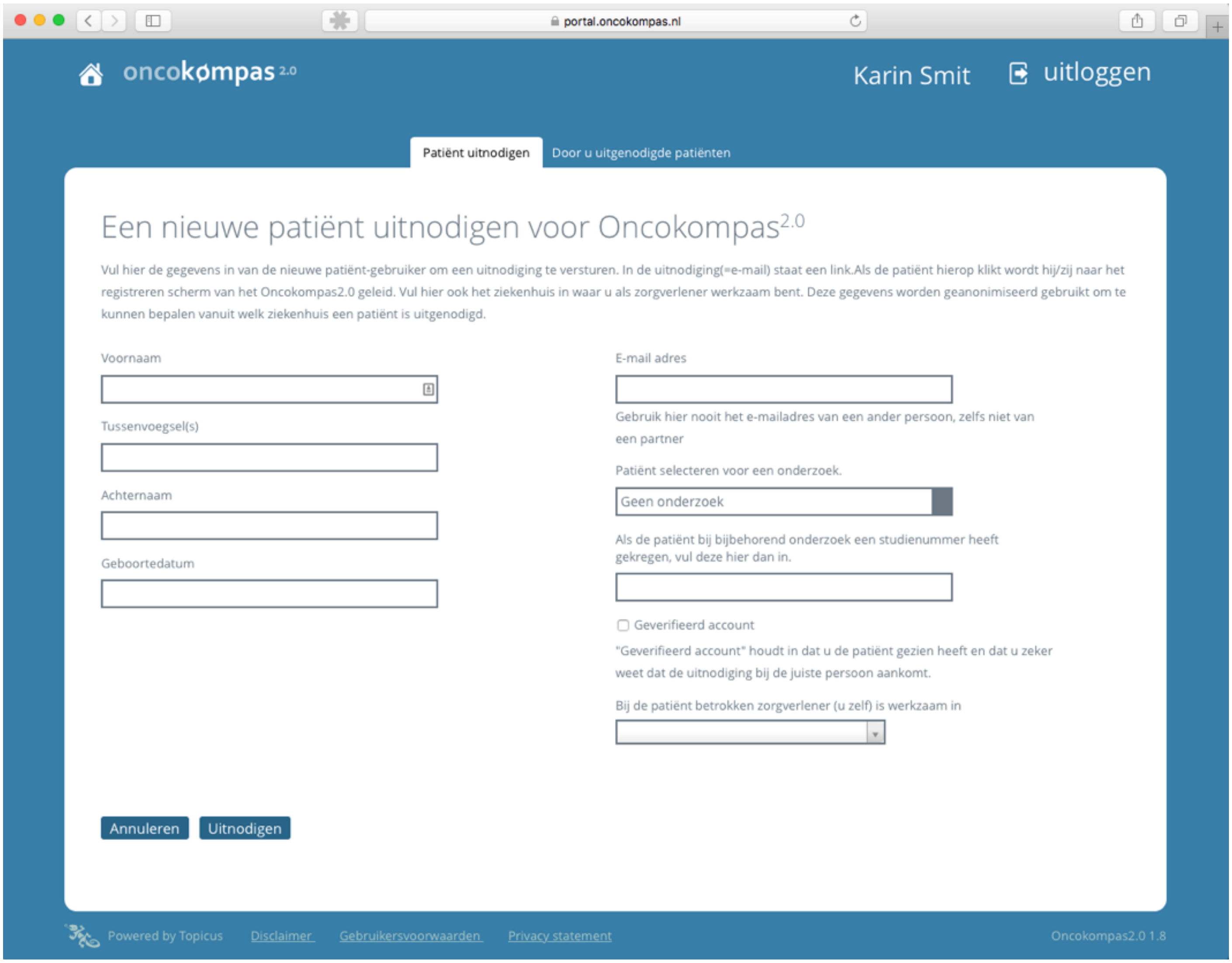

Stap 1: Meten

De deelnemer kan kiezen om het Oncokompas volledig te doorlopen door over alle onderwerpen vragen te beantwoorden of per onderwerp de vragen te beantwoorden.

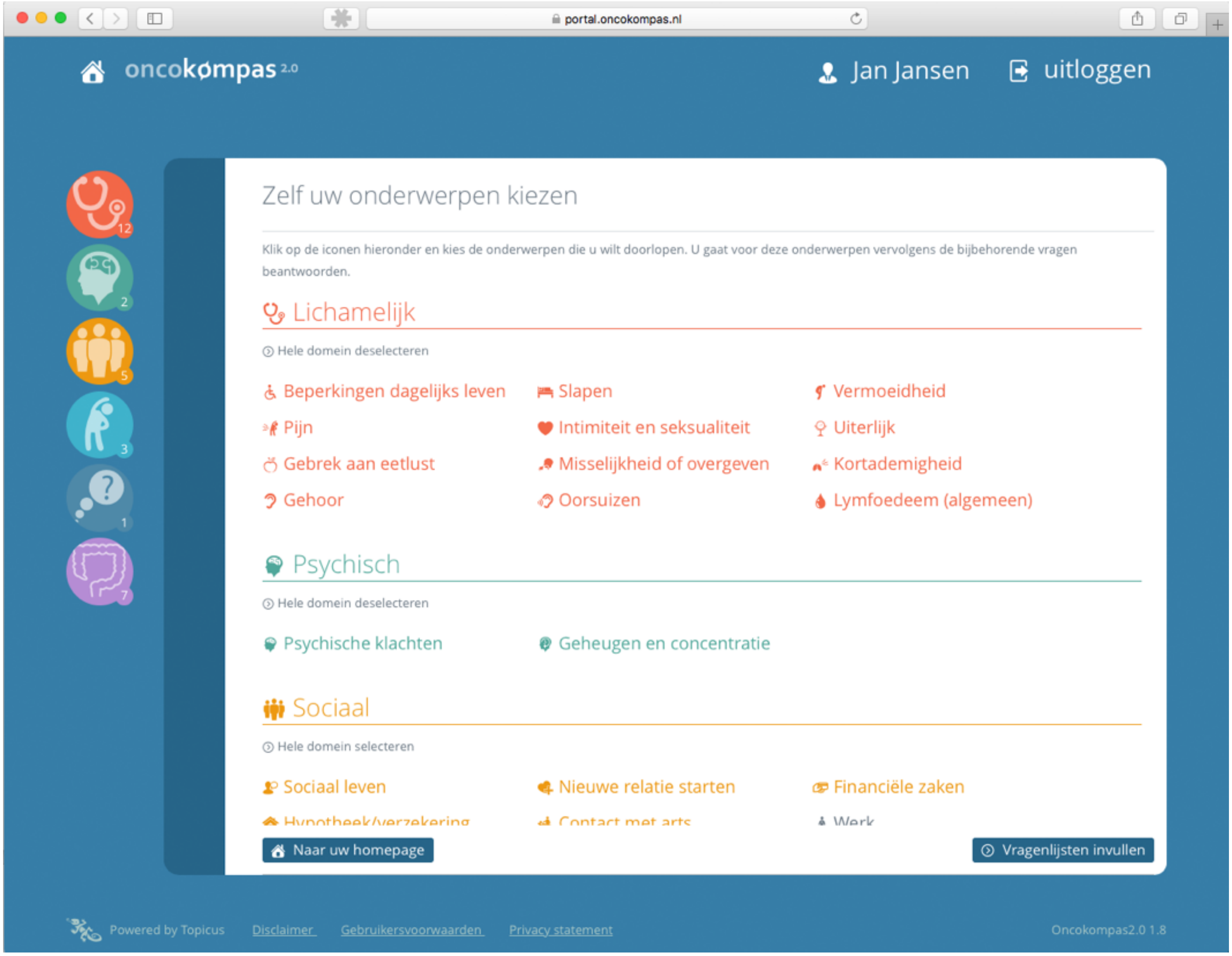

Overzicht van alle onderwerpen binnen het Oncokompas, verdeeld per domein van kwaliteit van leven.

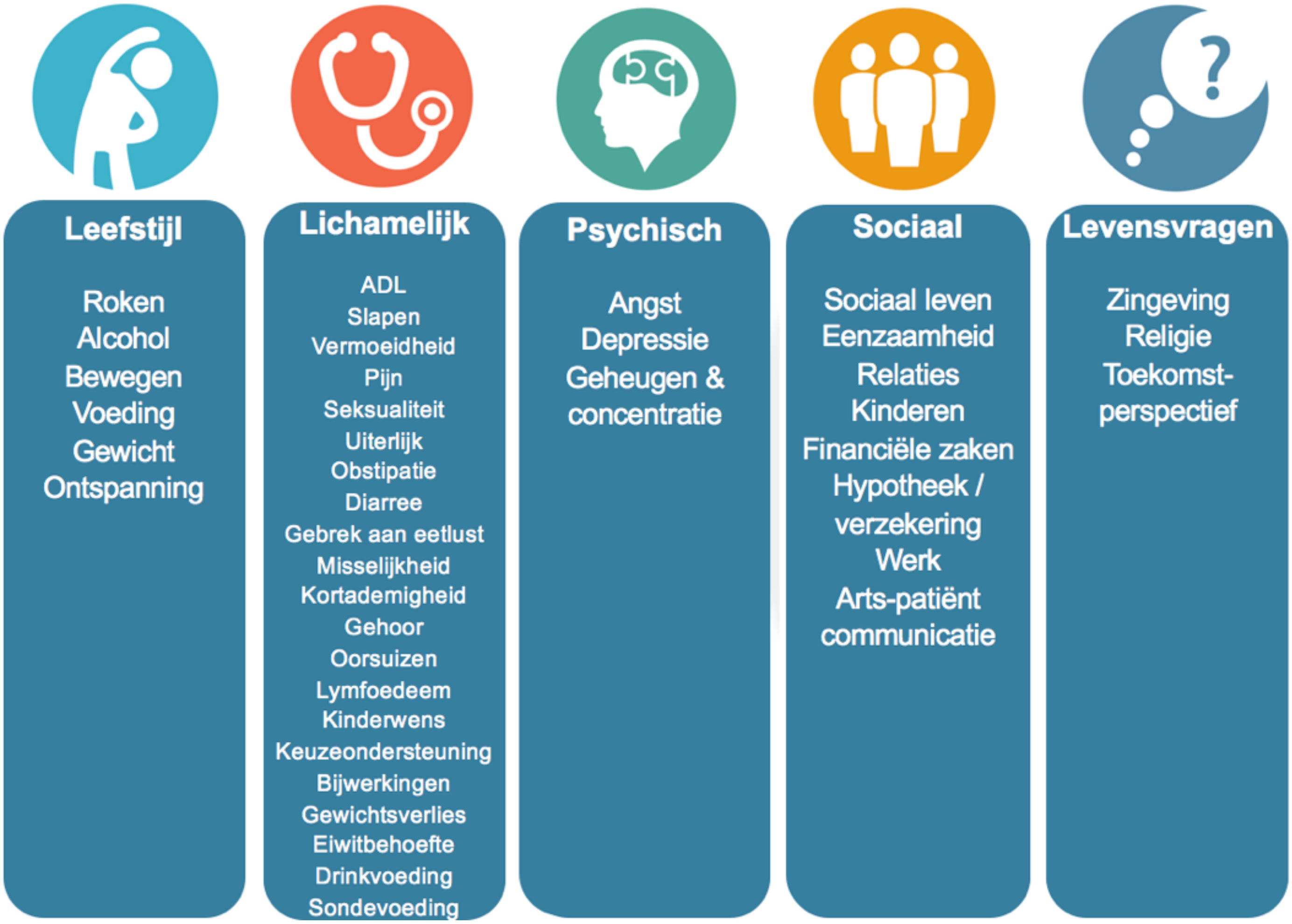

Voorbeeld van een vraag binnen een onderwerp.

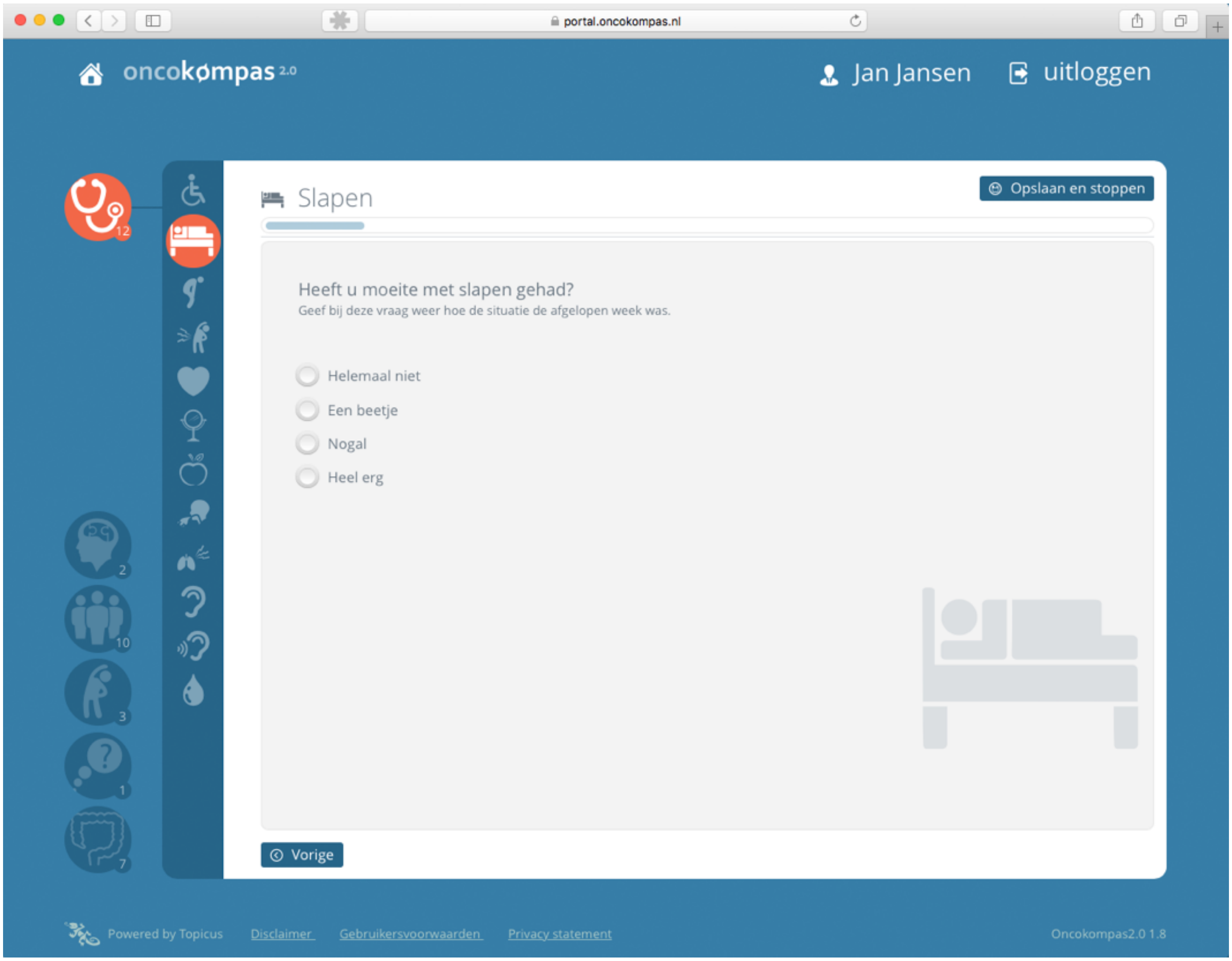

Stap 2: Weten

Na het doorlopen van de vragen volgt het welzijnsoverzicht. Op basis van de antwoorden die de deelnemer heeft gegeven, volgt per onderwerp een score op basis van stoplichtkleuren groen, oranje, rood.

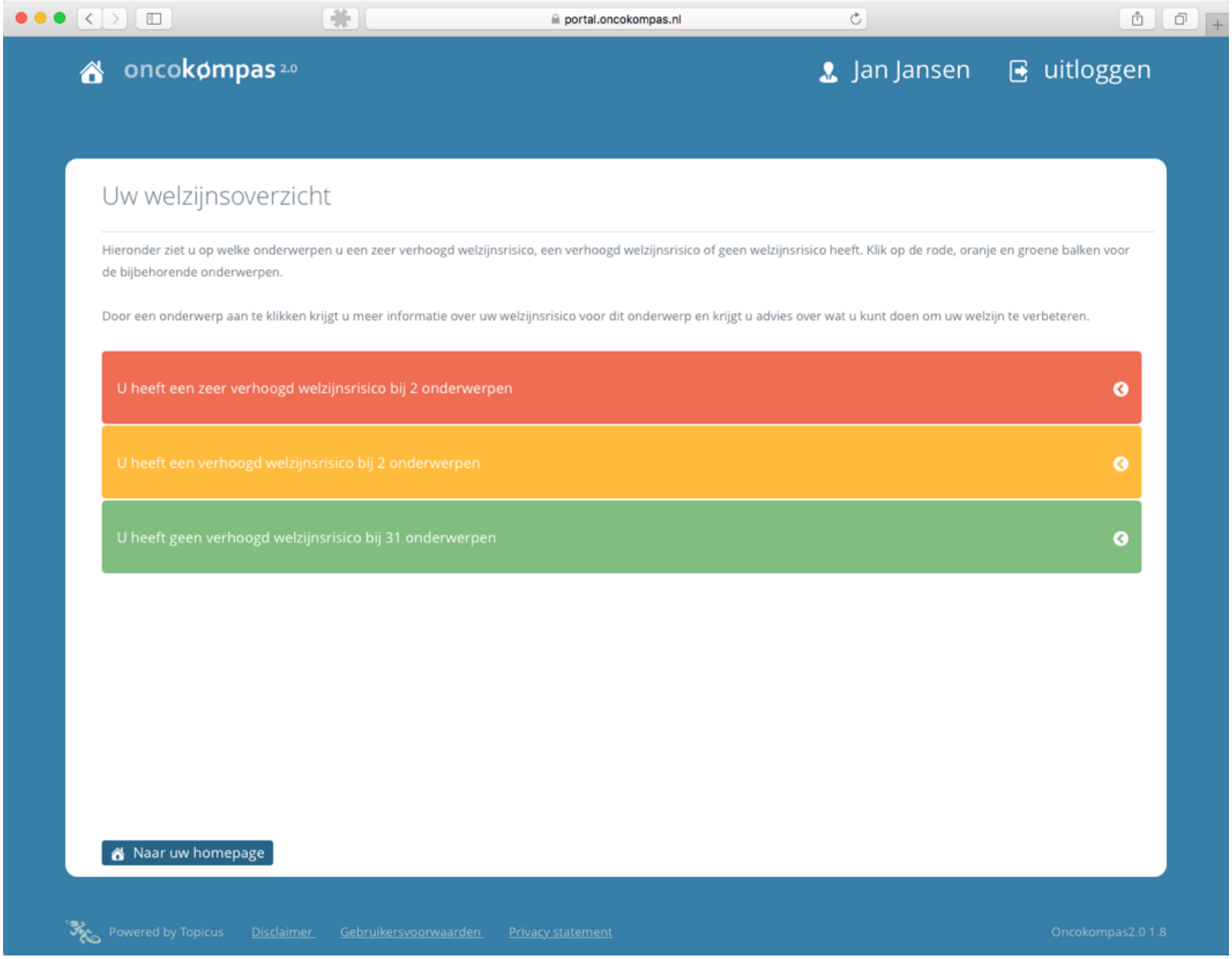

De deelnemer krijgt vervolgens per onderwerp informatie op maat, bijv. een inschatting van hoe lang dergelijke klachten kunnen aanhouden en hoe vaak de klacht voorkomt bij patiënten met kanker.

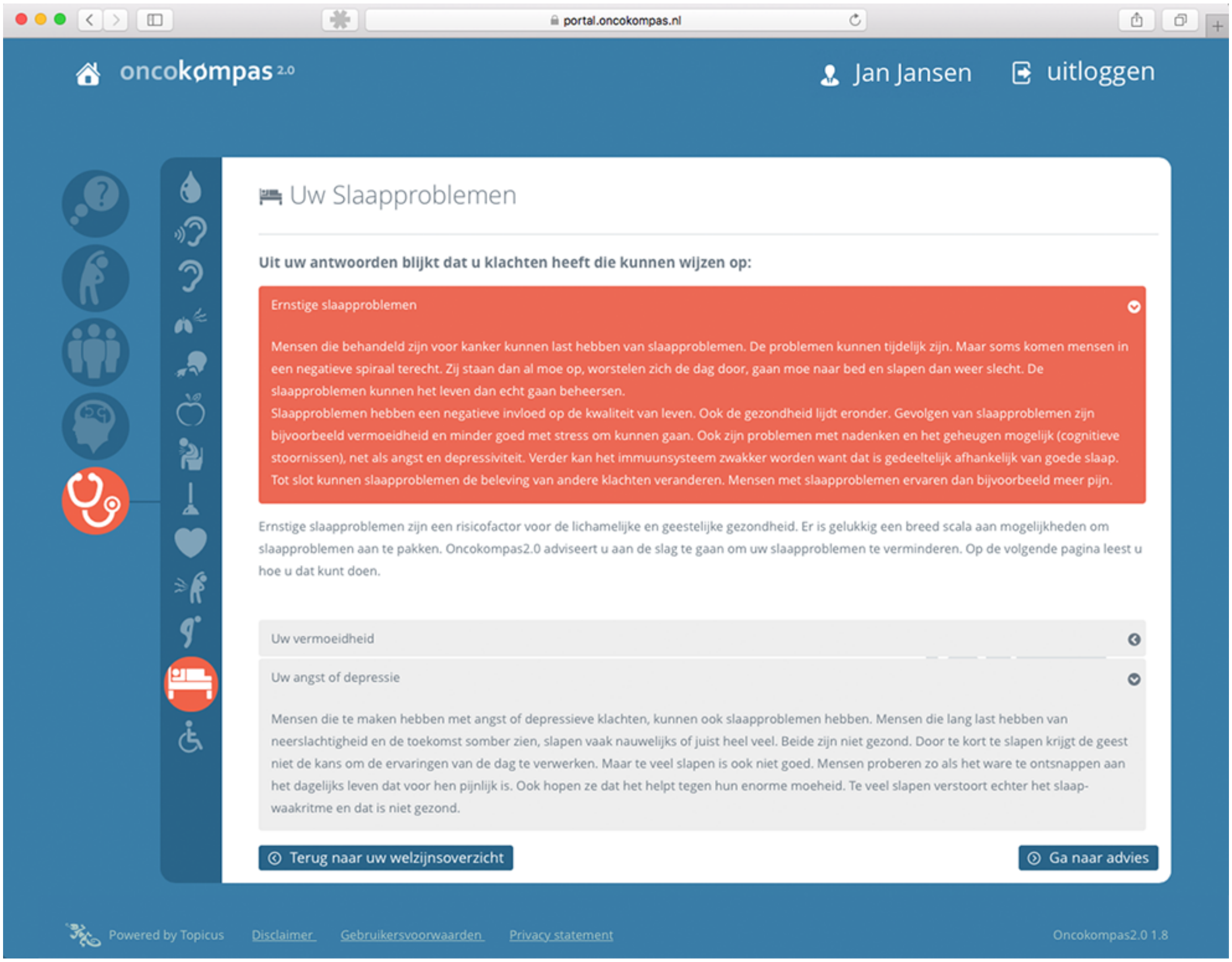

De deelnemer krijgt naast informatie per onderwerp, ook een persoonlijk gezondheidsadvies. Deze bestaat uit tips, maar ook bijv. brochures van KWF Kankerbestrijding of links naar het bijpassende onderwerp op www.kanker.nl.

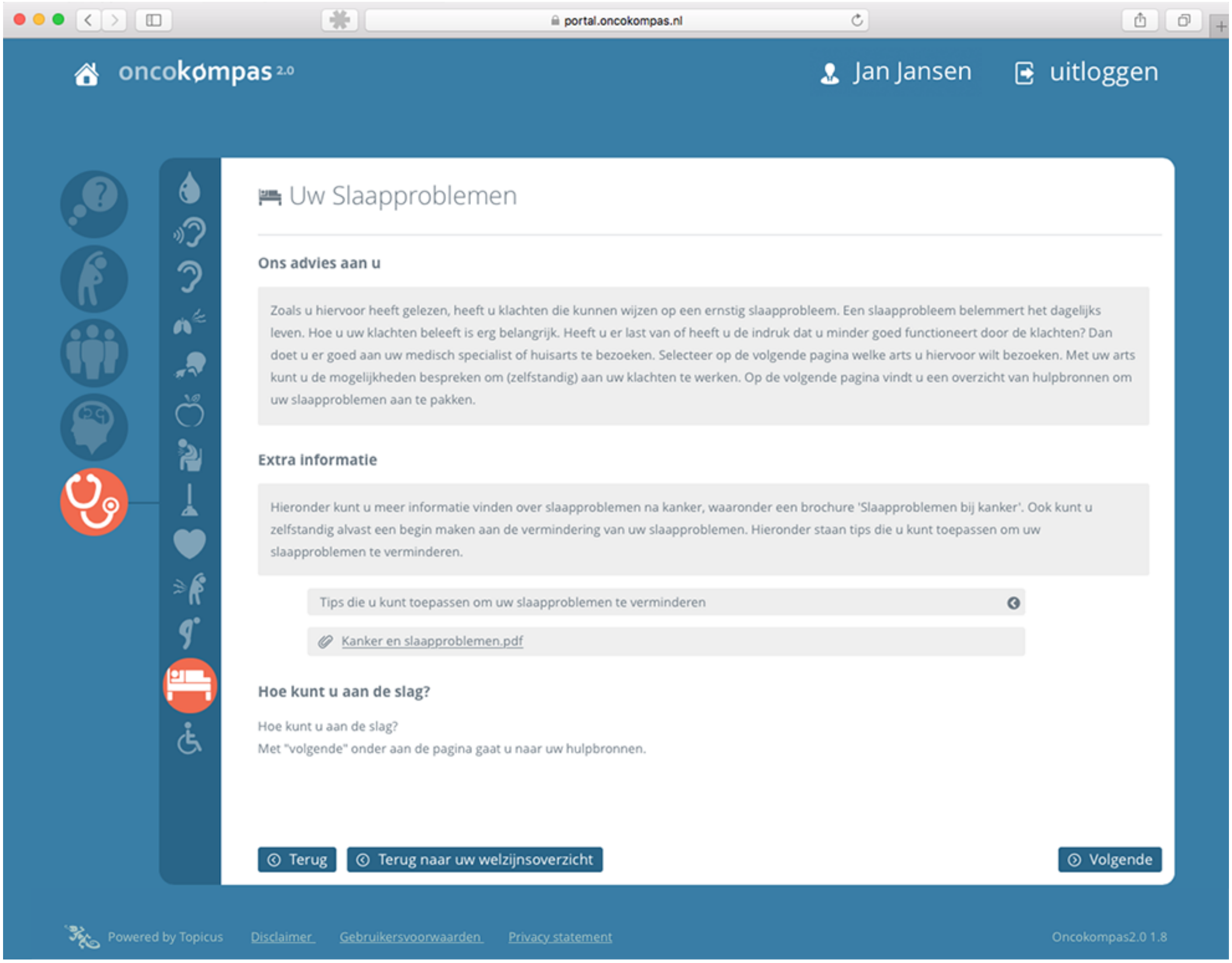

Stap 3: Doen

Op basis van de scores in het welzijnsdossier worden passende hulpbronnen aangeboden. Bij een rode score, zoals in onderstaand screenshot, wordt altijd eerst geadviseerd om naar een zorgprofessional bijv. (huis)arts, verpleegkundige of psycholoog) te gaan. Bij een oranje score worden eerst opties voor (online) zelfhulp weergegeven.

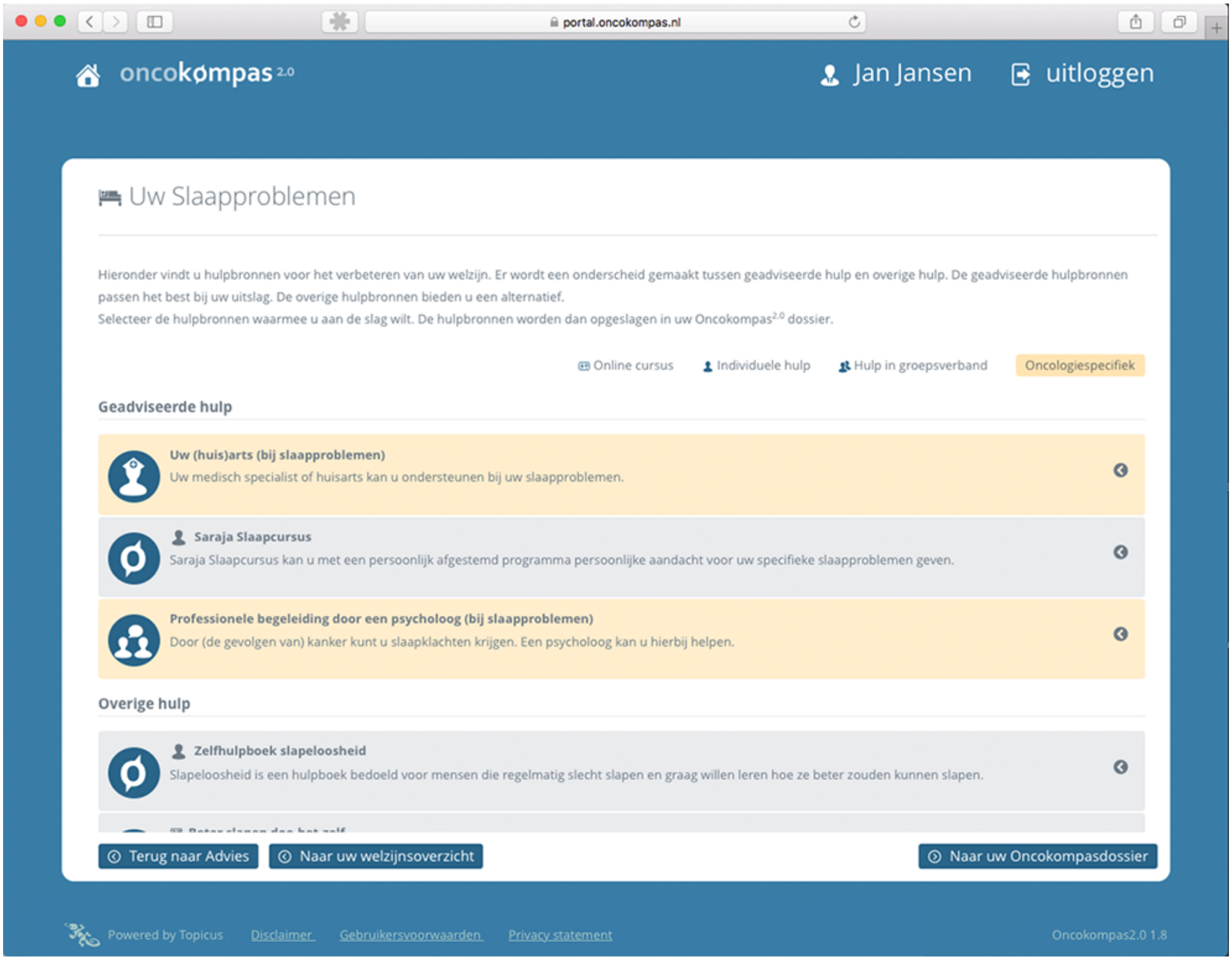

Als de deelnemer het Oncokompas meerdere keren heeft ingevuld, kan de deelnemer inzicht krijgen in zijn of haar ontwikkeling van kwaliteit van leven.

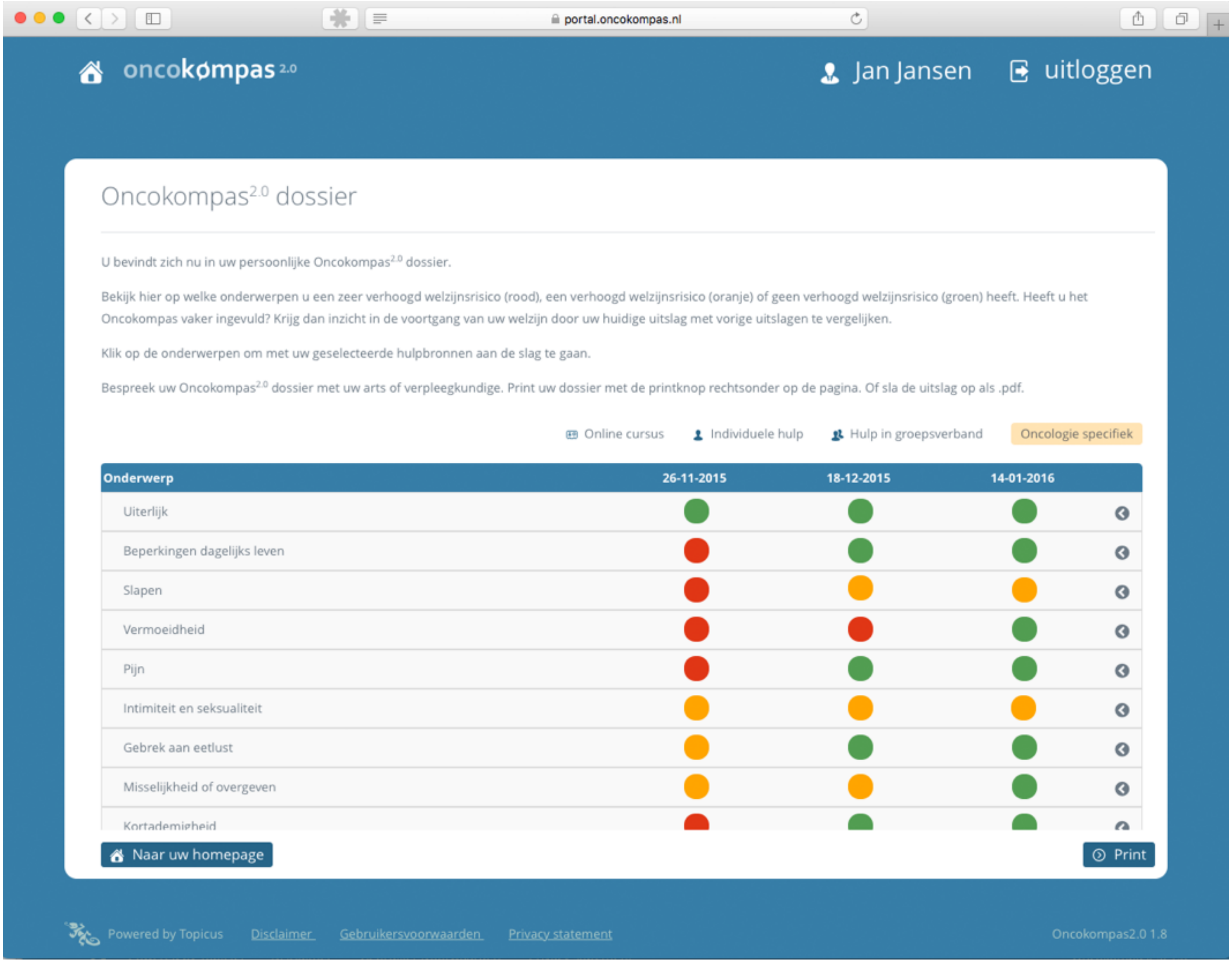

1. Wat spreekt u aan in (de omschrijving van) het Oncokompas?

2. Wat valt u tegen in (de omschrijving van) het Oncokompas?

3. Was u al bekend met het Oncokompas, alvorens het invullen van deze vragenlijst?

- ❍ Ja
- ❍ Nee

4. Wanneer verwacht u dat het gebruik van het Oncokompas voor uw patiënten in de richtlijnen wordt opgenomen?

- ❍ 2016
- ❍ 2017
- ❍ 2018
- ❍ Na 2018
- ❍ Nooit

5. In hoeverre bent u het eens met de volgende stellingen?

	helemaal mee oneens	mee oneens	noch mee oneens, noch mee eens	mee eens	helemaal mee eens
Als mijn zorginstelling het Oncokompas introduceert, ga ik me minder bij deze zorginstelling thuis voelen.	❍	❍	❍	❍	❍
Als mijn zorginstelling het Oncokompas introduceert, zal ik me minder betrokken voelen met deze zorginstelling.	❍	❍	❍	❍	❍

6. Stel dat het Oncokompas vandaag zou worden geïntroduceerd in uw zorginstelling. In hoeverre bent u het dan eens met de volgende stellingen:

	helemaal mee oneens	mee oneens	noch mee oneens, noch mee eens	mee eens	helemaal mee eens
Dan zou ik positief zijn tegen andere mensen over het Oncokompas.	❍	❍	❍	❍	❍
Dan zou ik andere mensen aanraden het Oncokompas in te zetten als ze het mij zouden vragen.	❍	❍	❍	❍	❍
Dan zou ik collega's en bekenden beïnvloeden om het Oncokompas in te zetten.	❍	❍	❍	❍	❍
Het Oncokompas zou niets voor mij zijn.	❍	❍	❍	❍	❍
Dan zou ik het Oncokompas niet inzetten.	❍	❍	❍	❍	❍

7. Stel dat uw zorginstelling het Oncokompas introduceert: In hoeverre bent u het eens met de volgende stellingen?

	helemaal mee oneens	mee oneens	noch mee oneens, noch mee eens	mee eens	helemaal mee eens
Ik zou me zorgen maken dat ik mijn baan kwijt raak.	❍	❍	❍	❍	❍
Ik zou het gevoel hebben dat mijn toekomstige carrière onzeker is.	❍	❍	❍	❍	❍
Ik zou me zorgen maken dat het belang van mijn werk en mijn autoriteit in het geding komen.	❍	❍	❍	❍	❍
Het zou waarschijnlijk zijn dat ik een positie met minder verantwoordelijkheid en autoriteit zou hebben in de organisatie.	❍	❍	❍	❍	❍
Ik zou verwachten dat ik een demotie (omgekeerde van een promotie) zou krijgen.	❍	❍	❍	❍	❍

8. Heeft u eventuele bedenkingen die u met ons wilt delen?

Dit is het einde van de vragenlijst.

Hartelijk dank voor uw deelname aan het onderzoek en het invullen van de vragenlijst.

Als u aanspraak wilt maken op de €10 vergoeding en/of kans wilt maken op de Apple Watch, Apple iPad of één van de vier VVV-bonnen t.w.v. 50 euro, kunt u op de volgende pagina's uw persoonlijke gegevens invullen.

Uw persoonlijke gegevens worden uitsluitend voor de uitbetaling van de €10 en/of u te informeren als u één van de prijzen heeft gewonnen. Uw gegevens zullen op geen enkele wijze worden gekoppeld aan de door u ingevulde antwoorden.

Noteer op de volgende pagina's uw gegevens als u aanspraak wilt maken op de €10 en kans wil maken op de prijzen. Als u enkel kans wilt maken op de prijzen, volstaat het om enkel uw e-mailadres in te vullen. U wordt dan geïnformeerd indien u in de prijzen valt.

Initialen:

Tussenvoegsels:

Achternaam:

E-mailadres:

Straat + huisnummer:

Postcode:

Woonplaats:

Datum invullen vragenlijst (dd/mm/jjjj):

Burgerservicenummer (BSN):

Geboortedatum (dd/mm/jjjj):

IBAN:

Kies uw bank (ten behoeve van de BIC):

- ABN AMRO Bank N.V.
- Achmea Retail Bank N.V.
- AEGON Bank N.V.
- Akbank N.V.
- Amsterdam Trade Bank N.V.
- Anadolubank N. V.
- Argenta Spaarbank N V
- ASN Bank N.V.
- Bank Insinger de Beaufort N.V.
- Bank Mendes Gans N.V.
- Bank Nederlandse Gemeenten N.V.
- Bank of America N.A.
- Bank of Scotland
- Bank of Tokyo-Mitsubishi (Holland)…
- BNP Paribas S.A.
- Citco Bank Nederland N.V.
- Citibank International plc
- Commerzbank AG, Kantoor Amsterdam
- Coöperatieve Centrale Raiffeisen-…
- Credit Europe Bank N.V.
- De Nederlandsche Bank
- Delta Lloyd Bank N.V.
- Demir-Halk Bank (Nederland) N.V.
- Deutsche Bank A.G.
- Dresdner Bank (Amsterdam branch)
- DSB Bank N.V.
- Economy Bank N.V., The
- F. van Lanschot Bankiers N.V.
- Friesland Bank N.V.
- GarantiBank International N.V.
- GE Artesia Bank
- Gilissen Bankiers N.V., Theodoor
- Hof Hoorneman Bankiers NV
- HSBC Bank Plc
- Indonesische Overzeese Bank, N.V. De
- ING Bank
- ING Belgium N.V.
- Intesa SanPaolo S.p.A.
- Isbank GmbH
- KAS Bank N.V.
- KBC Bank N.V.
- Knab
- Koçbank Nederland N.V.
- Korea Exchange Bank
- Landsbanki Islands hf
- Levob Bank N.V.
- Lloyds TSB Bank Plc
- Mizuho Corporate Bank Nederland N.V.
- Nederlandse Waterschapsbank N.V.
- NIBC Bank N.V.
- OHRA Bank NV
- Postbank (vanaf februari 2009: ING)
- Rabobank
- RegioBank
- Royal Bank of Scotland
- SNS Bank N.V.
- Société Générale
- Staal Bank N.V.
- Triodos Bank N.V.
- Volkswagen Bank GmbH NL
- Yapi Kredi Bank Nederland N.V.
- Anders, namelijk:

  ____

U mag mij benaderen voor toekomstig wetenschappelijk onderzoek:

- Ja
- Nee
