# Supplementary material for: Understanding Drivers of Resistance Toward Implementation of Web-Based Self-Management Tools in Routine Cancer Care Among Oncology Nurses: Cross-Sectional Survey Study
Source: J Med Internet Res. 2019 Dec 17;21(12):e14985. doi: 10.2196/14985 (PMC6938592; doi:10.2196/14985)
Supplement: Multimedia Appendix 1 [file jmir_v21i12e14985_app1.pdf]

Variabele	Description		Items	English translation	Original item	Scale (1-5)	Original Scale	Source	Comment	Factor loading	χ²(df), p-value	CFI[1]	TLI[1]	RMSEA	SRMR[3]	Pre CFA Cronbach's Alpha	Post CFA Cronbach's Alpha
INNOVATION-RELATED CHARACTERISTICS																	
Incompatibility	Incompatibilty with current working routines.	1	Ik denk dat het moeilijk zal zijn om het aanbieden van dergelijke online zelfmanagement hulpmiddelen in mijn bestaande werkwijze in te passen.	I think it will be difficult to incorporate offering online self-management tools into my existing working routine.	I think it is frustrating to fit this product into my existing usage patterns.	Strongly disagree - strongly agree		Gurtner (2014)		1.000	75.57 (3), p<0.001	1.000	1.000	0.000	0.000	0.595	0.589
		2	Het aanbieden van dergelijke online zelfmanagement hulpmiddelen is in strijd met mijn huidige manier van werken.	Offering online self-management tools conflicts with my existing way of working.	Using ... is in conflict with my current practices	Strongly disagree - strongly agree				0.587							
		3	Ik denk dat het aanbieden van dergelijke online zelfmanagement hulpmiddelen goed past in mijn dagelijkse routine. (R)	I think offering online self-management tools fits well into my existing daily working routine.	I think that using ... does not fit into my daily routine	Strongly disagree - strongly agree				0.640							
Complexity	The extent to which the HCP believes that using the technology requires effort (related to ease of use).	1	Dergelijke online zelfmanagement hulpmiddelen lijken mij moeilijk om te gebruiken.	Such online self-management tools seem difficult to use to me.	I find our SA-system easy to use	Strongly disagree - strongly agree	1-7 scale Strongly disagree – strongly agree	Schillewaert (2005)		1.000	219.2 (6), p<0.001	0.997	0.992	0.034	0.026	0.627	0.741
		2	Het lijkt mij makkelijk om dergelijke online zelfmanagement hulpmiddelen te laten doen wat ik wil. (R)	It seems easy to get such online self-management tools do what I want.	I find it easy to get the SA-system to do what I want it to do	Strongly disagree - strongly agree	1-7 scale Strongly disagree – strongly agree		Removed after CFA because of low factor loading	0.328							
		3	Dergelijke online zelfmanagement hulpmiddelen lijken mij lastig te begrijpen.	Such online self-management tools seem difficult to understand to me.	My interaction with our SA-system is clear and understandable	Strongly disagree - strongly agree	1-7 scale Strongly disagree – strongly agree			1.484							
		4	Ik moet erg nadenken bij het gebruik van dergelijke online zelfmanagement hulpmiddelen.	Using such online-selfmanagement tools requires a lot of my mental effort.	Using our SA-system does not require a lot of my mental effort	Strongly disagree - strongly agree	1-7 scale Strongly disagree – strongly agree			1.004							
Lack of value	Lack of value compared to existing alternatives.	1	Nieuwe online zelfmanagement hulpmiddelen bieden geen voordelen die al niet door andere, soortgelijke oplossingen geboden worden.	New online self-management tools offer no advantages that are not offered by other, similar solutions.	This product offers advantages that are not offered by competing products (r)	Strongly disagree - strongly agree	1-7 scale "Totally disagree - totally agree"	Heidenreich (2015)								0.448	0.465
		2	Nieuwe online zelfmanagement hulpmiddelen lossen problemen op die ook door bestaande tools kunnen worden opgelost.	New online self-management tools solve problems that can be solved with existing tools.	Removed: 2. This product is, in my eyes, superior to competing products (r) This product solves a problem that I cannot solve with competing products (r)	Strongly disagree - strongly agree	1-7 scale "Totally disagree - totally agree"										
Risk	Perceived risks about reliability and performance.	1	Ik heb er vertrouwen in dat dergelijke online zelfmanagement hulpmiddelen hetgeen zullen kunnen doen zoals beschreven. (R)	I am confident such online-selfmanagement tools will perform as described.	I am not confident that this product will perform as described	Strongly disagree - strongly agree	1-7 scale very confident-not confident at all	Heidenreich (2015)		1.000	71.95 (3), p<0.001	1.000	1.000	0.000	0.000	0.720	0.720
		2	Ik ben er niet zeker van dat dergelijke online zelfmanagement hulpmiddelen naar tevredenheid zullen werken.	I am not certain that such online self-management tools will work satisfactorily.	I am not certain that this product will work satisfactorily	Strongly disagree - strongly agree	1-7 scale certain-uncertain			1.077							
		3	Ik betwijfel of dergelijke online zelfmanagement hulpmiddelen betrouwbaar zijn in gebruik.	I doubt whether such online self-management tools are reliable in use.	I doubt whether the product is reliable in use	Strongly disagree - strongly agree				0.635							
Role ambiguity	The HCP's knowledge and understanding of what to do.	1	Ik voel me onzeker over hoe ik nieuwe online zelfmanagement hulpmiddelen effectief kan inzetten in mijn werk.	I feel uncertain about how to effectively use new online self-management tools in my job.	I feel certain about how to effectively use the SST	Strongly disagree - strongly agree	1-7 scale Strongly disagree – strongly agree	Meuter (2014)		1.000	20.89 (5), p=0.001	0.967	0.933	0.114	0.047	0.835	0.839
		2	Ik twijfel over hoe ik nieuwe online zelfmanagement hulpmiddelen op de juiste wijze moet gebruiken.	I am not sure how to use new online self-managemente tools properly.	I am NOT sure how to use the SST properly	Strongly disagree - strongly agree	1-7 scale Strongly disagree – strongly agree			1.054							
		3	Ik weet niet zeker wat van mij zal worden verwacht als ik nieuwe online zelfmanagement hulpmiddelen in zal zetten in mijn werk.	I do not know what is expected of me if I use new online self-management tools in my job.	I know what is expected of me if I use the SST	Strongly disagree - strongly agree	1-7 scale Strongly disagree – strongly agree			1.045							
		4	Ik denk dat de stappen in het proces om nieuwe online zelfmanagement hulpmiddelen te gebruiken mij niet duidelijk zullen zijn.	I think the steps in the process of using new online self-management tools are not clear to me.	The steps in the process of using the SST are clear to m	Strongly disagree - strongly agree	1-7 scale Strongly disagree – strongly agree			0.608							
		5	Ik verwacht dat er alleen vage aanwijzingen zullen zijn over het gebruik van nieuwe online zelfmanagement hulpmiddelen.	I believe there will only be vague directions regarding how to use new online self-management tools.	I believe there are only vague directions regarding ho	Strongly disagree - strongly agree	1-7 scale Strongly disagree – strongly agree			0.537							
Social pressure: institutional	Perceived coercive pressure from hospital management to offer online self-management tools to patients.	1	Ik denk dat mijn zorginstelling vindt dat we dergelijke online zelfmanagement hulpmiddelen zouden moeten aanbieden.	I think my healthcare institution believes we should offer such online self-management tools.	Our main customers that matter to us believe that we should use eSCM.	Strongly disagree - strongly agree	1-7 scale Strongly disagree – strongly agree	Liu (2010)		1.000	261.45 (6), p<0.001	0.992	0.977	0.064	0.029	0.670	0.790
		2	Wellicht verliest mijn zorginstelling een deel van de markt als ze dergelijke online zelfmanagement hulpmiddelen niet aanbieden.	My healthcare institution may lose market share if they don't offer such online self-management tools.	We may not retain our important customers without eSCM.	Strongly disagree - strongly agree	1-7 scale Strongly disagree – strongly agree		Removed after CFA because of low factor loading	0.432							
		3	Ik denk dat het ziekenhuismanagement er van overtuigd is dat we dergelijke online zelfmanagement hulpmiddelen moeten aanbieden.	I think hospital management is convinced we should offer such online self-management tools.	Our main suppliers that matter to us believe that we should use eSCM.	Strongly disagree - strongly agree	1-7 scale Strongly disagree – strongly agree			1.729							
		4	Ik verwacht dat het ziekenhuismanagement druk gaat uitoefenen om dergelijke online zelfmanagement hulpmiddelen aan te bieden.	I expect hospital management will exert pressure to offer such online self-management tools.	Our suppliers that are crucial to us hotly wish us to use eSCM.	Strongly disagree - strongly agree	1-7 scale Strongly disagree – strongly agree			1.246							
Social pressure: peers	Perceived coercive pressure from peers to offer online self-management tools to patients.	1	Ik denk dat mijn collega's vinden dat we dergelijke online zelfmanagement hulpmiddelen zouden moeten aanbieden.	I think my collegues believe we should offer such online self-management tools.	Our main customers that matter to us believe that we should use eSCM.	Strongly disagree - strongly agree	1-7 scale Strongly disagree – strongly agree	Liu (2010)		1.000	195.91 (3), p<0.001	1.000	1.000	0.000	0.000	0.745	0.742
		2	Ik denk dat de meeste verpleegkundigen vinden dat we dergelijke online zelfmanagement hulpmiddelen moeten aanbieden.	I think most nurses believe we should offer such online self-management tools.	Our main customers that matter to us believe that we should use eSCM.	Strongly disagree - strongly agree	1-7 scale Strongly disagree – strongly agree			1.194							
		3	Ik verwacht dat verpleegkundigen druk zullen uitoefenen om dergelijke online zelfmanagement hulpmiddelen aan te bieden.	I expect hospital management will exert pressure to offer such online self-management tools.	Our suppliers that are crucial to us hotly wish us to use eSCM.	Strongly disagree - strongly agree	1-7 scale Strongly disagree – strongly agree			0.688							
Social pressure: patients	Perceived coercive pressure from patients to offer online self-management tools to patients.	1	Ik verwacht dat mijn patiënten vinden dat we dergelijke online zelfmanagement hulpmiddelen zouden moeten aanbieden.	I expect that patients believe we should offer such online self-management tools.	Our main customers that matter to us believe that we should use eSCM.	Strongly disagree - strongly agree	1-7 scale Strongly disagree – strongly agree	Liu (2010)								0.615	0.603
		2	Ik denk dat er patiënten zijn die er van overtuigd zijn dat we dergelijke online zelfmanagement hulpmiddelen moeten aanbieden.	I think there are patients that are convinced we should offer such online self-management tools.	Our main suppliers that matter to us believe that we should use eSCM.	Strongly disagree - strongly agree	1-7 scale Strongly disagree – strongly agree										
STAFF-RELATED CHARACTERISTICS																	
Experience	Previous experience with internet, online selfmanagement tools, or other similar tools	1	In de dagelijkse praktijk maak ik op mijn werk nooit gebruik van het Internet.	I never use the Internet in in daily practice at work.		Strongly disagree - strongly agree	1-7 scale Strongly disagree – strongly agree		Removed due to factor loadings and Cronbach's Alpha, and to focus this scale to be on experience with online self-management tools	1.000	22.882 (3), p<0.001	1.000	1.000	0.000	0.000	0.334	
		2	In de dagelijkse praktijk bied ik patiënten vaak online zelfmanagement hulpmiddelen aan. (R)	I often offer online self-management tools to patients in daily practice.		Strongly disagree - strongly agree	1-7 scale Strongly disagree – strongly agree			9.097							
		3	In de dagelijkse praktijk maak ik vaak gebruik van de Lastmeter of een vergelijkbaar instrument. (R)	I often use the Distress Thermometer or similar tools in daily practice.		Strongly disagree - strongly agree	1-7 scale Strongly disagree – strongly agree		Removed due to factor loadings and Cronbach's Alpha, and to focus this scale to be on experience with online self-management tools	3.546							
Expertise	Confience in own capabilities regarding support of patients using online self-management tools. Can also be viewed als self-efficacy.	1	Ik verwacht goed in staat te zijn / ik ben goed in staat om patiënten te ondersteunen als het gaat om online zelfmanagement hulpmiddelen bij kanker.	I expect to be fully capable / I am fully capable supporting patients when it comes to online self-management tools for cancer.	I am fully capable of using the SST	Strongly disagree - strongly agree	1-7 scale Strongly disagree – strongly agree	Meuter (2014)		1.000	547.152 (6), p<0.00	1.000	1.007	0.000	0.009	0.860	0.852
		2	Ik heb vertrouwen in mijn vaardigheden om het gebruik van online zelfmanagement hulpmiddelen bij patiënten te ondersteunen.	I am confident in my ability to support the use of online self-management tools.	I am confident in my ability to use the SST	Strongly disagree - strongly agree	1-7 scale Strongly disagree – strongly agree			1.000							
		3	Het ondersteunen van patiënten ten aanzien van online zelfmanagement hulpmiddelen past goed binnen mijn mogelijkheden.	Supporting patients in the use of online self-management tools falls well within the scope of my abilities.	Using the SST is well within the scope of my abilities	Strongly disagree - strongly agree	1-7 scale Strongly disagree – strongly agree			0.730							
		4	Ik heb niet het gevoel dat ik gekwalificeerd ben om patiënten bij te staan in het gebruik van online zelfmanagement hulpmiddelen. (R)	I do not feel I am qualified for the task of supporting patients in the use of online self-management tools.	I do NOT feel I am qualified for the task for ordering a prescription refill with the SST	Strongly disagree - strongly agree	1-7 scale Strongly disagree – strongly agree			0.732							
Control variables																	
job description/task [CONTROL]	Tasks related to supporting self-management among patients.	1	Ik geef mijn patiënten meer inzicht in kwaliteit van leven en het verloop hiervan over de tijd.	I provide my patients with insights into quality of of life and its course over time.		Strongly disagree - strongly agree	1-5 scale (1) totally disagree, (2) disagree, (3) neither agree nor disagree, (4) agree, (5) totally agree	Fleuren (2014)		1.000	356.083 (10), p<0.0(	0.882	0.764	0.177	0.077	0.570	0.768
		2	Ik informeer mijn patiënten over klachten en de samenhang hiertussen.	I inform my patients about the coherence between symptoms.		Strongly disagree - strongly agree	1-5 scale (1) totally disagree, (2) disagree, (3) neither agree nor disagree, (4) agree, (5) totally agree			1.265							
		3	Ik voorzie mijn patiënten van advies op probleemgebieden.	I provide my patients with advice on problem areas.		Strongly disagree - strongly agree	1-5 scale (1) totally disagree, (2) disagree, (3) neither agree nor disagree, (4) agree, (5) totally agree			1.243							
		4	Ik raad mijn patiënten passende hulpbronnen aan.	I offer my patients appropriate resources.		Strongly disagree - strongly agree	1-5 scale (1) totally disagree, (2) disagree, (3) neither agree nor disagree, (4) agree, (5) totally agree			1.113							
		5	Ik stel mijn patiënten in staat om zelfstandig aan het herstel te werken.	I empower my patients to independently work on their recovery.		Strongly disagree - strongly agree	1-5 scale (1) totally disagree, (2) disagree, (3) neither agree nor disagree, (4) agree, (5) totally agree			0.746							
tendency to comply	Refers to HCP's adherence to guidelines and requests from leadership.	1	Wat betreft mijn manier van werken doe ik wat mijn leidinggevende mij vraagt te doen.	Regarding my way of working, I do what my superior asks me to do.	I try to do what the counselors ask me to do	Strongly disagree - strongly agree	1-7 scale 7-point Likert-type scales, anchored by never (1) and always (7).	Guo (2013)	Removed due to low Cronbach's Alpha							0.429	
		2	Wat betreft mijn manier van werken voer ik de richtlijnen zorgvuldig uit	Regarding my way of working, I thoughtfully execute the clinical guidelines.	I follow the recommended payment plan and guidelines	Strongly disagree - strongly agree	1-7 scale 7-point Likert-type scales, anchored by never (1) and always (7).		Removed due to low Cronbach's Alpha								
organizational commitment [CONTROL]	Refers to HCP's affective commitment to the organisation.	1	Ik ervaar de problemen van deze zorginstelling als mijn eigen problemen.	I really feel as if this healthcare organization's problems are my own.	I really feel as if this organization's problems are my own.	Strongly disagree - strongly agree	1-5 scale "5-puntsschaal, bestaande uit de antwoordcategorieën: 1 (in zeer zwakke mate op mij van toepassing), 2 (in zwakke mate op mij van toepassing), 3 (middelmatig op mij van toepassing), 4 (in sterke mate op mij van toepassing) en 5 (in zeer sterke mate op mij van toepassing). 2.4"	Meyer (1993)		1.000	631.413(10), p<0.00	0.983	0.966	0.090	0.032	0.847	0.842
		2	Ik heb het gevoel dat ik echt bij deze zorginstelling hoor.	I feel a sense of belonging to this healthcare organization.	I do not feel a 'strong' sense of belonging to my organization.(R)	Strongly disagree - strongly agree	1-5 scale "5-puntsschaal, bestaande uit de antwoordcategorieën: 1 (in zeer zwakke mate op mij van toepassing), 2 (in zwakke mate op mij van toepassing), 3 (middelmatig op mij van toepassing), 4 (in sterke mate op mij van toepassing) en 5 (in zeer sterke mate op mij van toepassing). 2.4"			1.683							
		3	Ik voel me emotioneel gehecht aan deze zorginstelling.	I feel emotionally attached to this healthcare organizaton.	I do not feel 'emotionally attached' to this organization.(R)	Strongly disagree - strongly agree	1-5 scale "5-puntsschaal, bestaande uit de antwoordcategorieën: 1 (in zeer zwakke mate op mij van toepassing), 2 (in zwakke mate op mij van toepassing), 3 (middelmatig op mij van toepassing), 4 (in sterke mate op mij van toepassing) en 5 (in zeer sterke mate op mij van toepassing). 2.4"			2.050							
		4	Ik voel me als 'een deel van de familie' in deze organisatie.	I feel like 'part of the family' at my organization.	I do not feel like 'part of the family' at my organization.(R)	Strongly disagree - strongly agree	1-5 scale "5-puntsschaal, bestaande uit de antwoordcategorieën: 1 (in zeer zwakke mate op mij van toepassing), 2 (in zwakke mate op mij van toepassing), 3 (middelmatig op mij van toepassing), 4 (in sterke mate op mij van toepassing) en 5 (in zeer sterke mate op mij van toepassing). 2.4"			2.023							
		5	Deze zorginstelling betekent veel voor mij.	This healthcare organization has a great deal of personal meaning for me.	This organization has a great deal of personal meaning for me.	Strongly disagree - strongly agree	1-5 scale "5-puntsschaal, bestaande uit de antwoordcategorieën: 1 (in zeer zwakke mate op mij van toepassing), 2 (in zwakke mate op mij van toepassing), 3 (middelmatig op mij van toepassing), 4 (in sterke mate op mij van toepassing) en 5 (in zeer sterke mate op mij van toepassing). 2.4"			1.883							
ORGANIZATION-RELATED CHARACTERISTCS																	
institutional orientation	Refers to organization's flexibility orientation, the desire for a focus on change or stability.	1	Mijn zorginstelling is zeer dynamisch en biedt ruimte voor een ondernemersgeest. Mensen zijn bereid hun nek uit te steken en risico's te nemen.	My healthcare organization is a very dynamic and entrepreneurial place. People are willing to stick their necks out and take risks.	Our organization is a very dynamic and entrepreneurial place. People are willing to stick their necks out and take risks.	Strongly disagree - strongly agree	1-7 scale Strongly disagree – strongly agree	Liu (2010)		1.000	405.1 (3), p<0.001	1.000	1.000	0.000	0.000	0.874	0.876
		2	Toewijding aan innovatie en ontwikkeling zijn kernelementen van mijn zorginstelling. Er wordt benadrukt voorloper te zijn met producten en diensten.	Dedication to innovation and development are core elements of my healthcare organization. There is an emphasis on being first with products and services.	The glue that holds our organization together is commitment to innovation and development. There is an emphasis on being first with products and services.	Strongly disagree - strongly agree	1-7 scale Strongly disagree – strongly agree			1.089							
		3	Binnen mijn zorginstelling is er een nadruk op groei via de ontwikkeling van nieuwe ideeën. Innovaties zijn belangrijk.	My healthcare organization emphasizes growth through developing new ideas. Innovations are important.	Our organization emphasizes growth through developing new ideas. Generating new products and services is important.	Strongly disagree - strongly agree	1-7 scale Strongly disagree – strongly agree			1.087							
support: institutional	The extent to which the HCP's organization explicitly encourages their employees to use online self-management tools.	1	Binnen mijn zorginstelling wordt de invoering van op de patiënt gerichte innovaties centraal gecoördineerd.	My healthcare organization centrally coordinates the introudction of patient focused innovations.		Strongly disagree - strongly agree	1-5 scale Strongly disagree - strongly agree									0.495	0.468
		2	Binnen mijn zorginstelling krijg ik als zorgverlener de ruimte om op de patiënt gerichte innovaties te introduceren.	My healthcare organization is a very dynamic and entrepreneurial place. People are willing to stick their necks out and take risks.		Strongly disagree - strongly agree	1-5 scale Strongly disagree - strongly agree										
support: technical	The HCP's perception about the extent to which HCPs receive support in case any operational assitence is needed for use of the online self-management technology.	1	Ik weet waar ik terecht kan als ik ondersteuning nodig heb bij online zelfmanagement hulpmiddelen voor patiënten.	I know where to turn to when I need any assistance with online self-management tools for patients.	I know where to turn to when I need any assistance with our SA-technology	Strongly disagree - strongly agree	1-7 scale Strongly disagree – strongly agree	Schillewaert (2005)		1.000	322.5 (3), p<0.001	1.000	1.000	0.000	0.000	0.787	0.790
		2	In mijn zorginstelling hebben we goede technische ondersteuning bij online zelfmanagement hulpmiddelen voor patiënten.	In my healthcare organization we get good technical support for online self-management tools for patients.	In my company we get good technical support for our SA-system	Strongly disagree - strongly agree	1-7 scale Strongly disagree – strongly agree			1.439							
		3	We hebben uitgebreide ondersteuning om problemen op te lossen met online zelfmanagement hulpmiddelen voor patiënten.	We have extensive support to help with problems related to online self-management tools for patients.	We have extensive support to help with problems related to our SA-system	Strongly disagree - strongly agree	1-7 scale Strongly disagree – strongly agree			1.275							
support: managerial	The extent to which HCP's immediate supervisors explicitly encourage their subordinates to use online self-management tools.	1	Ik word door mijn leidinggevende aangemoedigd om online zelfmanagement hulpmiddelen voor patiënten te gebruiken bij mijn werk.	I am continuously encouraged by my immediate supervisor to use online self-management tools for patients in my job.	I am continuously encouraged by my immediate supervisor (mijn leidinggevende) to use our SA-tool in my job	Strongly disagree - strongly agree	1-7 scale Strongly disagree – strongly agree	Schillewaert (2005)		1.000	531.5 (3), p<0.001	1.000	1.000	0.000	0.000	0.902	0.902
		2	Mijn leidinggevende ondersteunt uitdrukkelijk het gebruik van online zelfmanagement hulpmiddelen voor patiënten.	My immediate supervisor explicitly supports my using of online self-management tools for patients.	My immediate supervisor explicitly supports my using of our SA-system	Strongly disagree - strongly agree	1-7 scale Strongly disagree – strongly agree			1.038							
		3	Mijn leidinggevende gelooft oprecht in de voordelen van online zelfmanagement hulpmiddelen voor patiënten.	My immediate supervisor truly believes in the benefits of online self-management tools for patients.	My immediate supervisor truly believes in the benefits of our SA-system	Strongly disagree - strongly agree	1-7 scale Strongly disagree – strongly agree			0.799							
rate of adoption	The HCP's perception about the extent to which the HCP's organization integrates innovations into regular working processes.	1	Binnen mijn zorginstelling worden innovaties alleen binnen pilottrajecten ingezet en volgt geen structurele implementatie	Within my healthcare organization, innovations are only deployed within pilot projects, while subsequent structural implementation lacks.	Binnen mijn zorginstelling worden innovaties alleen binnen pilottrajecten ingezet en volgt geen structurele implementatie	Strongly disagree - strongly agree	1-5 scale (5) totally disagree, (4) disagree, (3) neither agree nor disagree, (2) agree, (1) totally agree		Removed due to low Cronbach's Alpha							0.306	
		2	Binnen mijn zorginstelling worden over het algemeen innovaties snel als onderdeel van reguliere processen geïmplementeerd. *	Within my healthcare organization, innovations are generally implemented quickly as part of the regular working processes.	Binnen mijn zorginstelling worden innovaties alleen binnen pilottrajecten ingezet en volgt geen structurele implementatie	Strongly disagree - strongly agree	1-5 scale (5) totally disagree, (4) disagree, (3) neither agree nor disagree, (2) agree, (1) totally agree		Removed due to low Cronbach's Alpha								
USER-RELATED CHARACTERISTICS																	
ability	The extent to which the HCP finds the innovation to be relevent for his/her patient.	1	Ik verwacht dat online zelfmanagement hulpmiddelen niet geschikt zijn voor mijn patiëntenpopulatie.	I expect online self-management tools are not suitable for my patients.	I think the innovation is relevant for my clients.	Strongly disagree - strongly agree	1-5 scale (5) totally disagree, (4) disagree, (3) neither agree nor disagree, (2) agree, (1) totally agree	Fleuren (2014)		1.000	531.5 (3), p<0.001	1.000	1.000	0.000	0.000	0.799	0.798
		2	Ik verwacht dat online zelfmanagement hulpmiddelen te belastend zijn voor mijn patiënten vanwege de vragenlijsten die al vanuit mijn zorginstelling worden afgenomen.	I expect online self-management tools are too burdensome for my patients because of the patient questionnaires my healthcare organization already uses.		Strongly disagree - strongly agree	1-5 scale (5) totally disagree, (4) disagree, (3) neither agree nor disagree, (2) agree, (1) totally agree			1.106							
		3	Ik verwacht dat online zelfmanagement hulpmiddelen te belastend zijn voor mijn patiënten vanwege hun ziekte en de bijbehorende behandeling.	I expect online self-management tools are too burdensome for my patients because of their ilness and accompanying treatment.		Strongly disagree - strongly agree	1-5 scale (5) totally disagree, (4) disagree, (3) neither agree nor disagree, (2) agree, (1) totally agree			1.240							
value	The extent to which the HCP expects the innovation to be valueable for his/her patient.	1	Ik verwacht dat mijn patiënten geen behoefte zullen hebben aan online zelfmanagement hulpmiddelen.	I expect my patients do not have a need for online self-management tools.		Strongly disagree - strongly agree	1-5 scale (5) totally disagree, (4) disagree, (3) neither agree nor disagree, (2) agree, (1) totally agree	Fleuren (2014)		1.000	106.617 (3), p<0.00	1.000	1.000	0.000	0.000	0.659	0.661
		2	Ik denk dat er al genoeg van dit soort dergelijke toepassingen beschikbaar zijn.	I think there are already enough tools of such available.		Strongly disagree - strongly agree	1-5 scale (5) totally disagree, (4) disagree, (3) neither agree nor disagree, (2) agree, (1) totally agree			0.839							
		3	Ik verwacht dat het merendeel van de patiënten geen gebruik zal maken van online zelfmanagement hulpmiddelen als ik deze aan zou bieden.	I expect most patients would not use online self-management tools if I would offer them.		Strongly disagree - strongly agree	1-5 scale (5) totally disagree, (4) disagree, (3) neither agree nor disagree, (2) agree, (1) totally agree			1.127							
ENVIRONMENT-RELATED CHARACTERISTICS																	
dynamics	The extent to which activities from other hospitals, companies, and organizations, technology, patient needs, and guideines, change frequently.		Geef aan hoe vaak de volgende aspecten in uw beleving ve	Indicate how often the following aspects regarding oncological care (in the Netherlands) change in your perception:				Homburg (2007)			86.306 (6), p<0.001	0.960	0.880	0.079	0.035	0.563	
		1	Producten en diensten van andere ziekenhuizen, organisaties en bedrijven	Products and services from other hospitals, organizations and companies		Almost never - very often	1-5 scale "very seldom" (1) and "very frequently" (5)		Combined with 3	1.000							0.476
		2	Technologische innovaties	Technological innovations		Almost never - very often	1-5 scale "very seldom" (1) and "very frequently" (5)		Combined with 4	0.741							0.283
		3	Behoeftes van patiënten	Needs of patients		Almost never - very often	1-5 scale "very seldom" (1) and "very frequently" (5)		Combined with 1	1.225							0.476
		4	Richtlijnen	Clinical guidelines		Almost never - very often	1-5 scale "very seldom" (1) and "very frequently" (5)		Combined with 2	0.834							0.283
Influence external stakeholders: insurer	Perceived coercive pressure from healthcare insurance companies to offer online self-management tools to patients.	1	Ik denk dat de ziektekostenverzekeraars ervan overtuigd zijn dat we online zelfmanagement hulpmiddelen moeten aanbieden.	I think that healthcare insurance companies are convinced we should offer online self-management tools.	Our main suppliers that matter to us believe that we should use eSCM.	Strongly disagree - strongly agree	1-7 scale Strongly disagree – strongly agree	Liu (2010)								0.808	0.807
		2	Ik verwacht dat ziektekostenverzekeraars druk zullen uitoefenen om online zelfmanagement hulpmiddelen aan te bieden.	I expect healthcare insurance companies will exert pressure to offer online self-management tools.	Our suppliers that are crucial to us hotly wish us to use eSCM.	Strongly disagree - strongly agree	1-7 scale Strongly disagree – strongly agree										
Influence external stakeholders: government	Perceived coercive pressure from the government to offer online self-management tools to patients.	1	Ik denk dat de overheid er van overtuigd is dat we online zelfmanagement hulpmiddelen moeten aanbieden.	I think the government is convinced we should offer such online self-management tools.	Our main suppliers that matter to us believe that we should use eSCM.	Strongly disagree - strongly agree	1-7 scale Strongly disagree – strongly agree	Liu (2010)								0.768	0.784
		2	Ik verwacht dat de overheid druk zal uitoefenen om online zelfmanagement hulpmiddelen aan te bieden.	I expect the government will exert pressure to offer such online self-management tools.	Our suppliers that are crucial to us hotly wish us to use eSCM.	Strongly disagree - strongly agree	1-7 scale Strongly disagree – strongly agree										
RESISTANCE BY STAFF																	
postponement		1	Op dit moment overweeg ik nog niet om dergelijke online zelfmanagement hulpmiddelen in te zetten.	I am not considering offering online self-management tools at this moment.		Strongly disagree - strongly agree		Heidenreich (2015)		1.000	330.0 (3), p<0.001	1.000	1.000	0.000	0.000	0.850	
passive resistance (formerly postponement)		2	Ik stel mijn beslissing over het gebruik van dergelijke online zelfmanagement hulpmiddelen op dit moment nog even uit.	For the moment I postpone my descision on the use of such online self-management tools.		Strongly disagree - strongly agree				1.221							0.852
		3	Ik ben geïnteresseerd in het gebruik van dergelijke online zelfmanagement hulpmiddelen, maar ik ga het op dit moment nog niet inzetten.	I am interested in using such online self-management tools, but will not use them at this time.		Strongly disagree - strongly agree				0.934							
rejection	An active evaluation on part of the HCP, which results in a strong disinclination to adopt online self-management tools.	1	Het is zeer onwaarschijnlijk dat ik dergelijke online zelfmanagement hulpmiddelen in de nabije toekomst ga inzetten.	It is unlikely that I use such online self-management tools in the near future.	It is unlikely that I use ... in the near future	Strongly disagree - strongly agree		Gurtner (2014)	Based on correlations and modification indices (and theory and practice) combined into one scale: active resistance	1.000	188.5 (3), p<0.001	1.000	1.000	0.000	0.000	0.640	
active resistance (formerly rejection + opposition)	An active evaluation on part of the HCP, which results in a strong disinclination to adopt online self-management tools. Combined with the extent to which HCPs are convinced that the innovation is unsuitable and decide to launch an attack.	2	Dergelijke online zelfmanagement hulpmiddelen zijn niets voor mij.	Such online self-management tools are not for me.	...is not for me	Strongly disagree - strongly agree			Based on correlations and modification indices (and theory and practice) combined into one scale: active resistance	1.503							0.884
		3	Ik ga dergelijke online zelfmanagement hulpmiddelen niet inzetten.	I will not use such online self-management tools.	I don't need ...	Strongly disagree - strongly agree			Based on correlations and modification indices (and theory and practice) combined into one scale: active resistance	1.481							
opposition	The extent to which HCPs are convinced that the innovation is unsuitable and decide to launch an attack.	1	Ik zal negatief zijn tegen andere mensen over dergelijke online zelfmanagement hulpmiddelen.	It is likely that I say negative things about such online self-management tools to other people.	It is very likely that I say negative things about ... to other people	Strongly disagree - strongly agree		Gurtner (2014)	Based on correlations and modification indices (and theory and practice) combined into one scale: active resistance	1.000	555.8 (3), p<0.001	1.000	1.000	0.000	0.000		
		2	Ik zou andere mensen aanraden dergelijke online zelfmanagement hulpmiddelen niet in te zetten als ze het mij zouden vragen.	It is very likely that I will advise other people not to use such online self-management tools if they ask me.	It is very likely that I will advise other people not to use ... if they ask me.	Strongly disagree - strongly agree			Based on correlations and modification indices (and theory and practice) combined into one scale: active resistance	1.113							
		3	Ik zou collega's en bekenden beïnvloeden om dergelijke online zelfmanagement hulpmiddelen niet in te zetten.	It is very likely that I will influence colleagues and acquaintances not to use such online self-management tools.	It is very likely that I will influence my friends not to use ...	Strongly disagree - strongly agree			Based on correlations and modification indices (and theory and practice) combined into one scale: active resistance	1.036							
intention to adopt	The HCP's intention to adopt online self-management tools when they are introduced.		Stel dat nieuwe online zelfmanagement hulpmiddelen vandaag zouden worden geïntroduceerd in uw zorginstelling:	Imagine that new online self-management tools would be introduced within your healthcare organization today:				Heidenreich (2015)	Removed due to inconsistent factor loadings, and in hindsight a completely different construct than resistance		239.9 (3), p<0.001	1.000	1.000	0.000	0.000	0.770	0.772
		1	Hoe waarschijnlijk is het dat u dergelijke online zelfmanagement hulpmiddelen zou aanbieden aan patiënten?	How likely do you feel it is that you would offer such online self-management tools to your patients?	How likely do you feel it is that you would purchase this product?	Very unlikely - very likely	1-4 scale 1. Very unlikely—Very likely 2. Highly improbable—Highly probable 3. Impossible—Possible 4. Unimaginable—Imaginable		Removed due to inconsistent factor loadings, and in hindsight a completely different construct than resistance	1.000							
		2	Hoe waarschijnlijk is het dat u dit gaat doen binnen drie maanden?	How probable is it you would do that within three months?		Very unlikely - very likely	1-4 scale 1. Very unlikely—Very likely 2. Highly improbable—Highly probable 3. Impossible—Possible 4. Unimaginable—Imaginable		Removed due to inconsistent factor loadings, and in hindsight a completely different construct than resistance	2.368							
		3	En binnen een jaar?	And within a year?		Very unlikely - very likely	1-4 scale 1. Very unlikely—Very likely 2. Highly improbable—Highly probable 3. Impossible—Possible 4. Unimaginable—Imaginable		Removed due to inconsistent factor loadings, and in hindsight a completely different construct than resistance	1.936							
